# Provenance and deposition of a lithified volcanic-rich layer (VRL-5.5) at 5.5 Ma from Central Apennines (Italy)

**DOI:** 10.1038/s41598-023-33256-2

**Published:** 2023-04-27

**Authors:** Davide Potere, Gianluca Iezzi, Vittorio Scisciani, Anna Chiara Tangari, Manuela Nazzari

**Affiliations:** 1 Dipartimento di Ingegneria & Geologia, Università G. d’Annunzio, Via dei Vestini 30, 66100 Chieti, Italy; 2 Istituto Nazionale di Geofisica e Vulcanologia, Via di Vigna Murata 605, 00143 Rome, Italy; 3 Dipartimento di Scienze Psicologiche, della Salute e del Territorio, Università G. d’Annunzio, Via dei Vestini 30, 66100 Chieti, Italy

**Keywords:** Volcanology, Stratigraphy

## Abstract

Two slightly lithified volcanic rich layers (VRL) (former tephra) SVT-2 (San Vittorino) and CAC (Castiglione a Casauria) were sampled from two distinct post-evaporitic Messinian stratigraphic sections (Abruzzo, Central Italy). They crop only few tens of km apart and are predominantly massive, although some specimens show sedimentary structures. Both VRLs were investigated for the first time by field, mesoscopic, X-ray powder diffraction (XRPD), transmission optical microscopy (TOM), scanning electron microscopy (SEM), bulk composition, electron-microprobe analysis (EMPA) and quantitative textural attributes by image analysis. The XRPD analysis detects the presence of a glass phase, plus few (< 2 area %) magmatic-like feldspars, clinopyroxene and biotite and stratigraphically variable sedimentary minerals such as calcite, dolomite, illite and montmorillonite (from 0 to 40 area %). The 2D image analysis performed on SEM microphotographs reveals that both sections are composed of very fine glass shards, magmatic minerals are never isolated, whilst the carbonate crystals mainly fill voids among volcanic particles. Both these VRLs have identical rhyolitic glass compositions that closely overlap with those of previously-studied coeval and stratigraphically related sections occurring in the northern Apennine region and dated as 5.5 Ma. The 2D textural features of glassy particles (length, width, aspect ratio, grain-size distribution, *M*_*Z*_ , *σ*_*i*_, *SK*_*i*_, *K*_*G*_ and roundness) in both SVT-2 and CAC sections are very similar and also close to the northern section of Camporotondo (Marche region). The outcomes provided here indicate that SVT-2 and CAC sections represent the southernmost distal deposits of the same large eruption that occurred about 5.5 Ma (VRL-5.5). They result from distal fallout of tephra through seawater, occasionally remobilised under low energy and localised conditions, especially in the uppermost part of the CAC section. All the VRL-5.5 rocks are probably related to a very large eruption that occurred in the Carpathian-Pannonian magmatic district. The analytical protocols used in this study can be useful to investigate other ancient volcanic-rich layers, corresponding to lithified tephra.

## Introduction

Explosive eruptions produce pyroclastic materials in an instantaneous geological time; their fall and flow deposits furnish markers for stratigraphic, volcanological, paleoclimatic, paleoenvironmental, and archaeological studies^1–4^. Airfall (loose) deposits are mainly composed of fine particles (lapilli 2 – 64 and ashes < 2 mm), although coarser components (blocks or bombs > 64 mm) may be present, mantling areas ranging from local to continental scales, whilst flows are un-sorted and form deposits (ignimbrites) with area extensions from few to hundreds of km^2^ (super-eruptions) from their vents^1,2,5,6^. These deposits are easily eroded by subaerial processes, but more readily preserved under submarine and lacustrine environments^3,7–9^. Extended fall-like volcanic-rich layers (VRL) are frequently the unique witness of past volcanic and magmatic events and systems, with completely obliterated centre of emission^1,3,4,10–13^.

The fragmentation of magmas produces several juvenile pyroclastic components, such as glass, minerals and pores; in addition cognate, accessory and accidental crystals and lithics can be also embedded^1,5,8,11,14^. If unconsolidated, i.e. loose, they are largely and broadly referred as tephra^1,15^, either undisturbed or having been subjected to further mechanical processes leading to secondary deposition^3,13,16–19^, as well as chemically altered by exogenous sedimentary processes^1,4,20^. The term “reprocessed” has been proposed for a sub-category of secondary deposits, i.e. syn-eruptive deposits made of particles directly produced by an eruption, but finally emplaced by a non-volcanic process^19^. An airfall tephra can be thus considered primary when its thickness is preserved immediately after deposition^3,21,22^. These tephra are characterised by an internal stratigraphy and sharp contacts at the top and bottom^3^, while remobilised tephra are thicker than the primary equivalent at the same distance from the source; frequently they include fractions of other “in situ” materials, such as non-volcanic or altered pre-existing volcanic clasts and bioclasts. In addition, they usually display gravity-driven sedimentary structures, soft-sediment deformation plus bioturbation and presence of fossils, as well as modification of grain size distribution (GSD) and grain shapes^3,7,16,18,22^.

These features can be readily recognised if tephra layers are well exposed as a function of distance from centre of emission and then moving from proximal to distal areas. By contrast, the possible re-deposition processes can mask or alter primary features, especially for very ancient tephra deposits^3,18^. Non-remobilised primary tephra may show variations in original thickness due to differential preservation and the variable degree of compaction from load^9^. Pyroclastic and epiclastic terms are also commonly used in the literature to distinguish between volcanic materials directly deposited after magma fragmentation and ejection *versus* those weathered, eroded and redeposited after primary depositions^10,14,16,23^. In addition to pyroclastic and epiclastic materials, effusive volcanic processes can produce autoclastic and hyaloclastic material^14^, but their distributions are limited to hundreds of meters from emission centres.

**Figure 1:**
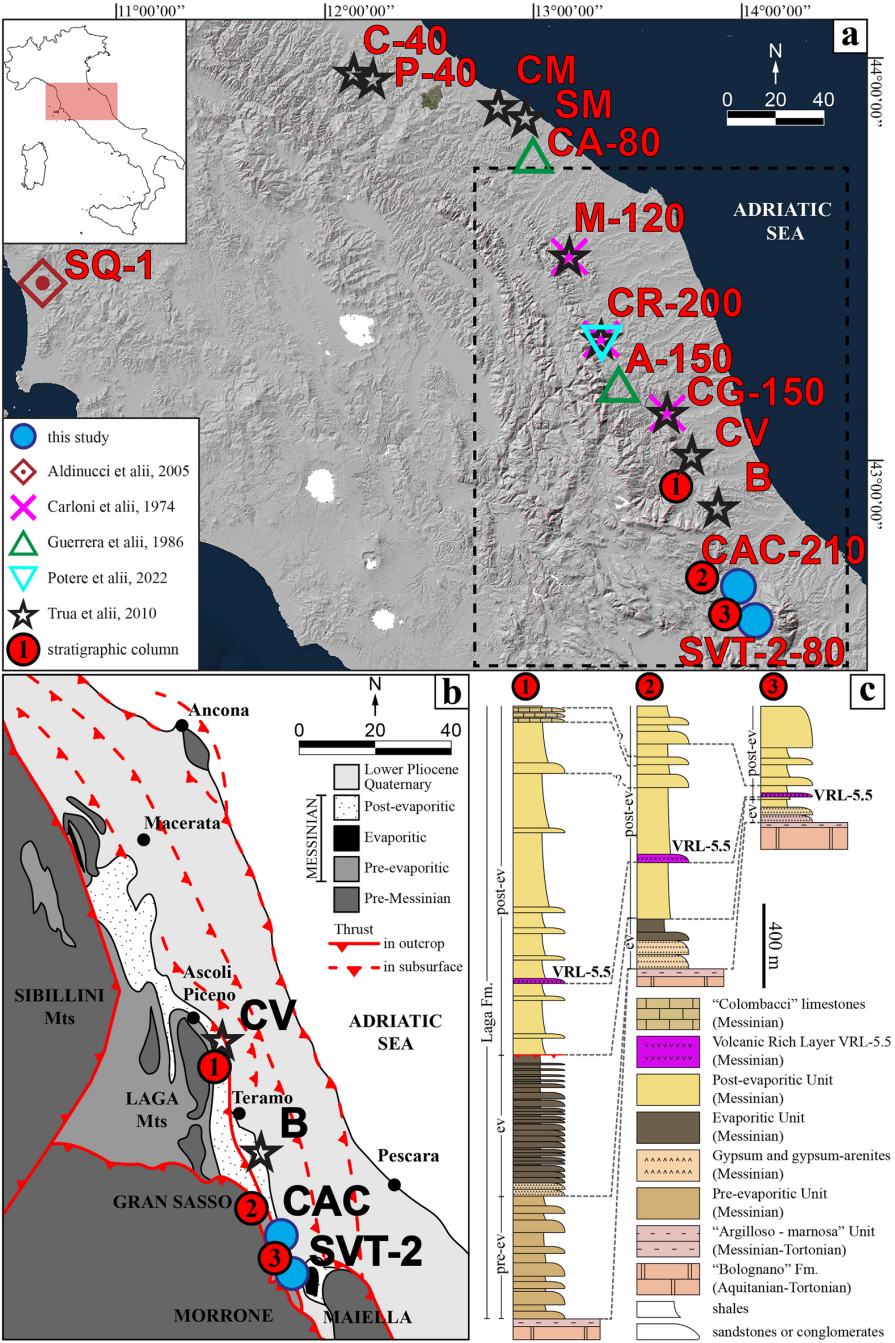
(**a**) Location of the samples of the VRL-5.5 deposits from previous studies and the two SVT-2 and CAC investigated in this study, with thickness in centimetres. (**b**) Schematic structural model of outer Central Apennines (dashed rectangle in (**a**) with the principal sedimentary units and tectonic elements (modified from Patruno et al. 2021). (**c**) Representative stratigraphic columns of the Laga basin from north to south: 1) Civitella del Tronto, 2) Brittoli-Queglia and 3) Morrone (N-E) (modified from Centamore et al. 2006). The acronyms are: A Amandola; B Bisenti; C Campea; CA Calcinelli; CAC Castiglione a’ Casauria; CG Colle Gallo; CM Casteldimezzo; CR Camporotondo; CV Civitella del Tronto; M Maccarone; P Piavola; SQ Serredi quarry; SM Santamarina; SVT-2 San Vittorino.

To further extend the spectra of previous processes and terminology, here we investigate two old and slightly lithified VRLs. Their induration does not allow them to be referred to as tephra, and prevents classical analysis by sieving methods. Both these VRLs are interbedded in the Messinian post-evaporitic sequence and crop out in the Abruzzo (close to San Vittorino and Castiglione a Casauria villages) (Table S, Fig. 1). These two sections have been characterised by field and mesoscopic rock observations, bulk chemical composition, X-ray powder diffraction (XRPD), transmission optical microscopy (TOM), scanning electron microscopy (SEM) and micro-chemical characterisation by electron probe micro-analysis (EPMA). These two investigated layers fall within the same stratigraphic interval of the ones previously studied and tabulated in Table S2^24–33^. The new attained results extend previous findings and reinterpret the origin and deposition mechanism of the VRL. The sampling procedures and analytical methods used here can be applied to other lithified volcanic-rich horizons, since they allow a complete chemical and textural characterization, useful for lateral correlation and for the interpretation of the mechanisms of transport and emplacement of distal pyroclasts.

## Geological setting, stratigraphy, and sampling

The two analysed VRLs are exposed in the eastern sector of the Apennines chain, where they are intercalated in the sedimentary sequence of the Neogene Adriatic foredeep-foreland basin (Fig. 1b, c). From the Miocene, the deep Adriatic foredeep basin developed in front of the advancing Apennines fold-and-thrust belt and was filled by a thick succession exceeding 7000 m in thickness of deepwater turbidites^34^. In this area, the oldest foredeep deposits (Messinian in age) consist of siliciclastic turbidites of the Laga Formation (Fm.)^35,36^ and overlay the pre-orogenic carbonate substratum (Fig. 1b, c). The two CAC and SVT-2 layers are both present in the upper part of the Laga Fm., thus being coeval and stratigraphically equivalent to other VRL sites cropping out further north (Fig. 1b, c). They are included in the post-evaporitic member (p-ev1 unit in the new classification^37^) (Fig. 1c). Similar stratigraphically-equivalent VRL-5.5 (see below), investigated in previous studies, were first dated at 5.4–5.5 Ma in the Maccarone section^28^ (Fig. 1a) and further refined to about 5.532 ± 0.0074 Ma^33^. In the Laga basin, the oldest foredeep depocenter was infilled by at least 3000 m of Messinian turbidites in the present-day Laga Mountain area^38^. Successively, this depocenter was laterally shifted to the east (i.e., east of the Montagnone-Montagna dei Fiori Mts) and minor subsidence also occurred in the southern area. The latter experienced reduced subsidence when compared with the northern sector^36,39^.

The studied area is located in the southern portion of the Messinian Laga basin. The two investigated stratigraphic columns are less than 20 km away (Fig. 1a, b). In these two sites, the late Miocene stratigraphy starts from the bottom with few meters of hemipelagic marls (Argilloso-marnosa Unit rich in *Orbulina* sp.) overlaid by gypsum-arenites with euxinic marls; the gypsum rich deposits pass upward to shales interbedded with the volcanic-rich horizon (post-evaporitic unit, Fig. 1c). The upper portion of the post-evaporitic succession consists of shales with thick-bedded sandstones, the limestones of the Colombacci Formation^27^ and channelized conglomeratic beds that prevail at the top. A generalized thickness reduction and facies thinning of the entire Messinian section towards the south have been documented^36,39^, indicative of a progressive termination of the Laga basin.

**Figure 2:**
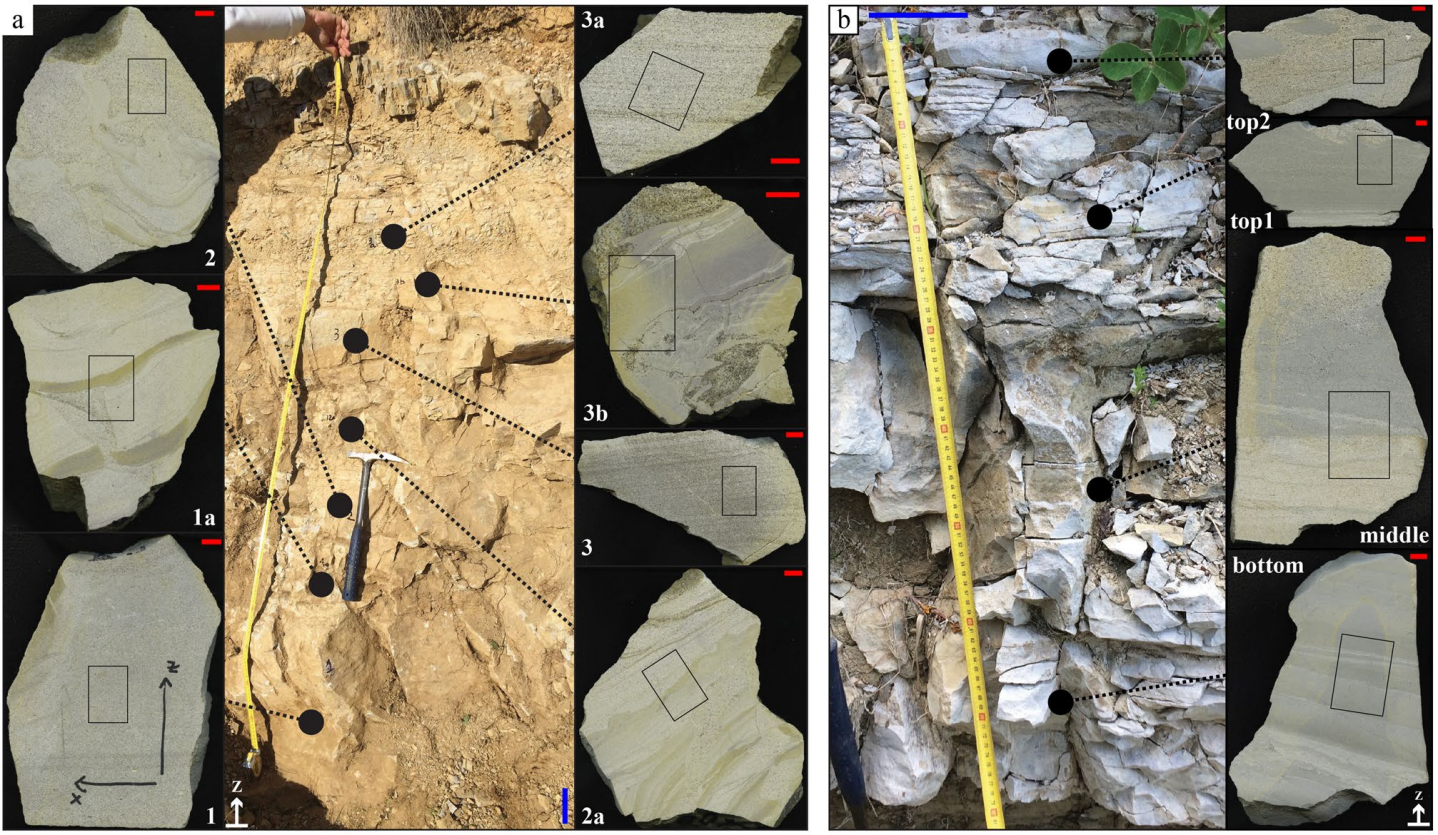
Stratigraphy of the (**a**) CAC section (Castiglione a’ Casauria, Abruzzo) and (**b**) SVT-2 section (San Vittorino, Sant'Eufemia, Abruzzo), with the position of the collected oriented samples (left) used to prepare the mesoscopic, polished samples near perpendicular to bedding (right); black rectangles indicate the positions of thin sections (displayed in Fig. 3a, b respectively). The complete CAC section is reported in Fig. S1. We sampled this lower, thicker, and more massive bed representative of the entire section The red and blue bars correspond to 1 and 10 cm, respectively.

The two VRL sections SVT-2 and CAC measure about 80 and 210 cm, respectively (Figs. 2, S1). The SVT-2 consists of fractured greyish rocks, and it is made up of several main massive sub-layers from which four oriented samples have been collected (Fig. 2b). The CAC is mainly composed of light brown to greyish poorly fractured rocks and contains several sub-horizons, again massive, according to field observations (Figs. 2a, S1). We sampled fifteen specimens from bottom to top (Fig. S1); the lowermost seven of these samples were considered for petrographic investigations (Fig. 2a). Each of the four SVT-2 and seven CAC oriented samples were cut and polished to expose their surfaces normal to the bedding of the layers (Fig. 2). The polished surfaces allow the observation of dm to mm-sized features (Fig. 2), possible phaneritic phases plus pores, as well as to select the most representative portions for thin section preparation.

## Analytical methods

### XRPD

The four SVT-2 plus the fifteen CAC specimens were first analysed by X-ray powder diffraction, following the procedure reported extensively in previous studies^40,41^. About 100 g of each sample (Fig. 2) was first milled in a semi-automatic grinder under acetone, to obtain homogeneous coarse powders with grains of few hundreds of µm. Around 20–30 mg of coarse grained and powder per sample was then further ground with an agate pestle and a mortar, again under acetone, for about ten minutes. The final and homogeneous powders have average grain sizes of a few µm. These nineteen fine powders were gently mounted in the central hole of a cylindrical zero background sample holder (diameter of 15 mm and depth of 0.44 mm), made of an oriented Si monocrystalline wafer. This Si nominal zero background allows the presence of non-crystalline phases to be highlighted, showing classical shoulder at about 2θ of 20–30°; otherwise, the background is flat apart from the presence of crystalline phases^40,42^. These fine powders were gently mounted in the hole avoiding, as much as possible, crystallite orientations, sample surface roughness and poorly dense sample packing^43^.

The prepared samples were scanned with a D-5005 Bruker–Siemens diffractometer, operating in the θ-2θ Bragg–Brentano configuration. It is equipped with a Cu X-ray source, a Ni filter and a scintillator detector. The spectra were all acquired from 4 to 82° of 2θ, with a step-scan of 0.02° and a counting time of 8 s per step^43,44^. Three further sub-samples (see below) were also analysed with a Rigaku Miniflex II benchtop diffractometer, with a Cu X-ray source, a Ni filter and a scintillator detector. These samples were loaded on a flat glass sample-holder to acquire XRPD patterns from 3 to 60° of 2θ, at the speed of 2° of 2θ per minute with a step of 0.02°. The measured Bragg reflection were first identified with the Bruker’s DIFFRAC.EVA phase ID software and then further refined with FIZ-Karlsruhe’s ICSD Desktop Windows interface (Inorganic Crystal Structure Database). The crystalline standards from ICSD that better reproduce both positions and relative intensities of measured Bragg reflections were then selected.

#### Bulk chemical composition

The two levels richest in pyroclasts and depleted in carbonates per sites (SVT-2-bottom, SVT-2-top1, CAC-1a, CAC-3b), as observed qualitatively by XRPD, were selected to determine bulk chemical compositions (Table S3). Whole-rock chemical compositions were determined by Activation Laboratories LTD (Ontario, Canada), according to the analytical protocols code 4B1 total Digestion ICP, code 4F-CaCO_3_ IR, code 4F-FeO titration, code 4F-H_2_O+-gravimetric and code 4LITHO lithium metaborate/tetraborate fusion-ICP and ICP-MS packages. A more detailed description of these analyses is reported in the ACTLABS web-site: https://actlabs.com/geochemistry/lithogeochemistry-and-whole-rock-analysis/lithogeochemistry/ and https://actlabs.com/geochemistry/lithogeochemistry-and-whole-rock-analysis/carbon-and-sulphur/^45^. These analyses quantified the amount of major oxides, principal volatiles species (H_2_O^−^, H_2_O^+^, CO_2_ and S^tot^), the FeO/Fe_2_O_3_ ratio and the loss on ignition (LOI) (Table S3).

#### SEM and EPMA

**Figure 3:**
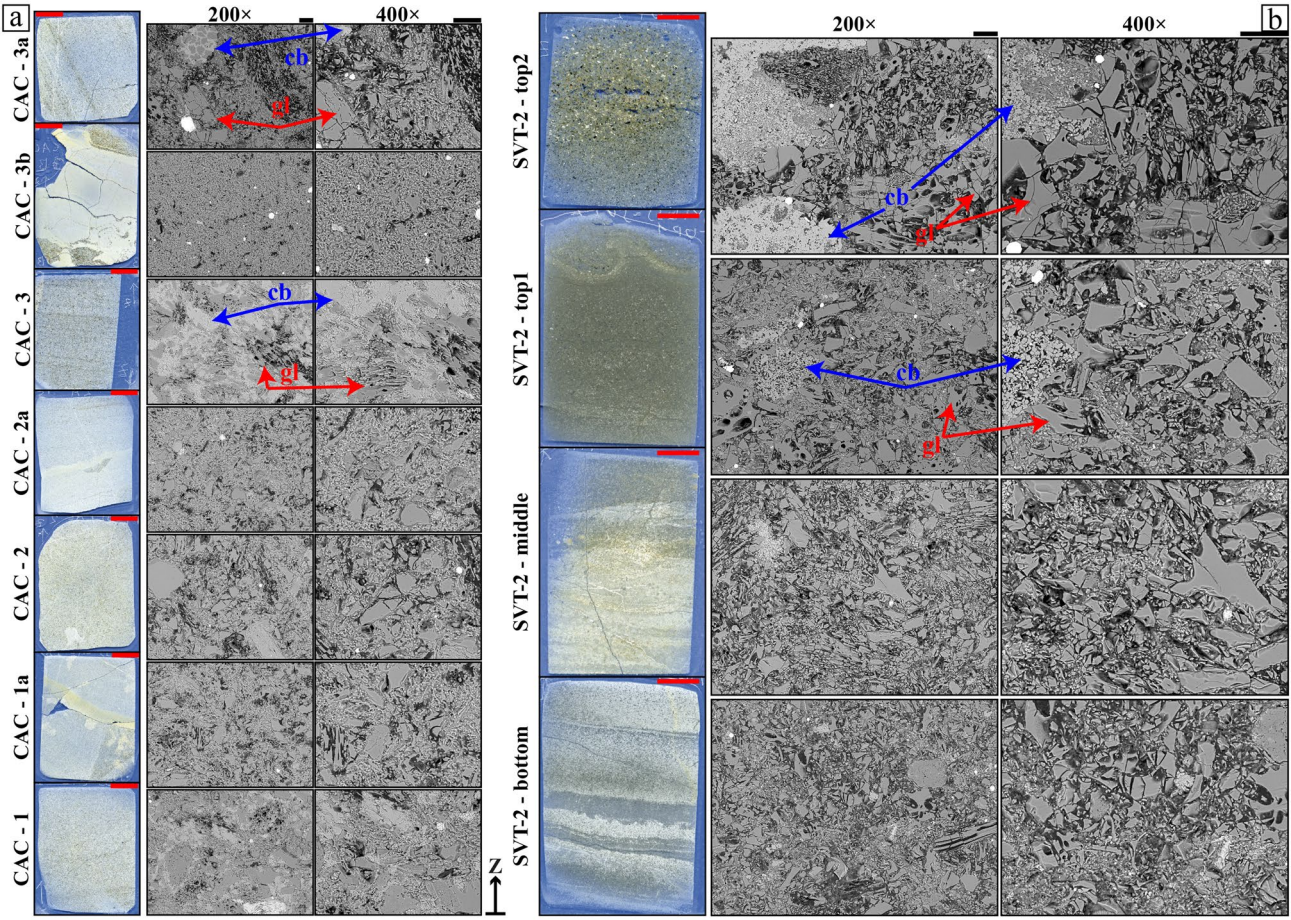
(**a**) CAC (Castiglione a’ Casauria, Abruzzo) and (**b**) SVT section (San Vittorino, Sant'Eufemia, Abruzzo) polished thin sections (black rectangles in Fig. 2) with representative BS-SEM microphotographs, showing the salient textural features along the stratigraphic sequence. Red and black bars are 1 cm and 50 µm, respectively, while blue arrow (cb) and red arrow (gl) indicates carbonates and glassy phases. The glass shards show blocky shapes with some curvilinear edges corresponding to bubble walls.

The four selected samples from SVT-2 and the lowermost seven from CAC, mounted on polished thin sections (Fig. 3), were analysed at the Istituto Nazionale di Geofisica e Vulcanologia (INGV) of Roma (Italy) with the EMPA Jeol-JXA8200, which is equipped with both EDS and WDS detectors and five spectrometers. The Field Emission Gun-SEM is a Jeol-JSM6500F equipped with an EDS detector. EPMA allowed us to determine the micro-chemical composition of the phases, using a voltage and current of 15 kV and 10 nA, respectively; a defocused electron beam with a spatial resolution of 10–15 µm^2^ was used on glassy grains, the most abundant phase^45,46^. The standard used for the micro-analyses was an augite from Dumfriesshire, provided by MAC (Micro-Analysis Consultants). Fifteen back scattered SEM (BS-SEM) images were collected per sample, amounting to 165 digital micro-photographs. Their magnification ranges from 100 to 1600×, but the sizes of pyroclastic components were more suitably captured at magnifications ranging between 100 and 400× (Figs. 3, S2).

#### Image analysis

Image analysis was carried out using Image-Pro Plus 6.0 software (http://www.mediacy.com/imageproplus) on 66 microphotographs acquired by SEM in back-scattered electron mode: 3 per sample at 100× and 200×, in the upper, central and lower area. Particles parameters were determined on thin section at 200×, while abundance (area %) of crystals, glass and carbonate on 100× images. Each microphotograph was first calibrated assigning it an appropriate spatial scale, then converted to 256 grey and finally filtered to enhance the shape and contours of particles^44,47,48^. Glass, carbonates and crystals were automatically segmented as a function of their ranges of grey tones, as schematically summarised in Fig. S2. When required, the measurement of a single object was manually performed, before automatically counting it^48^. Each object is represented by its corresponding equal-area ellipse to quantify its major axis, minor axis, aspect ratio, area and the angle between the major axis and the horizontal. According to resolution of pyroclastic objects and representative SEM magnifications, a threshold was applied such to exclude any object with a major and minor axis < 10 and < 5 µm, respectively. In addition, the values of the perimeter and the roundness were quantified per clast. Once defined,

$$rondness=\frac{{perimeter}^{2}}{4*\pi *area}$$

circular objects will have roundness of 1, while other shapes will have a roundness > 1 [modified from^49,50^].

Since all the collected rocks are lithified, 3D measurements, like for loose pyroclasts, are precluded. Consequently, size distributions were determined with 2D image analysis data^44,51^. The statistical parameters of grain-size distribution were calculated, after converting the grain-size to a phi-scale as $\Phi =-{\mathrm{log}}_{2}D$, where *D* is the length of the major axis in mm. These statistical parameters correspond to the classical granulometric indexes: mean grain size (M_Z_); inclusive graphic standard deviation (σ_i_); inclusive graphic skewness (Sk_i_); graphic kurtosis (K_G_)

$${M}_{Z}=\frac{{\Phi }_{16}+{\Phi }_{50} +{\Phi }_{84}}{3}$$

$${\sigma }_{i}=\frac{{\Phi }_{84}-{\Phi }_{16}}{4}+\frac{{\Phi }_{95}-{\Phi }_{5}}{4}$$

$${SK}_{i}=\frac{{\Phi }_{84}+{\Phi }_{16}-2{\Phi }_{50}}{2*({\Phi }_{84}-{\Phi }_{16})}+\frac{{\Phi }_{95}+{\Phi }_{5}-2{\Phi }_{50}}{2*({\Phi }_{95}-{\Phi }_{5})}$$

$${K}_{G}=\frac{{\Phi }_{95}-{\Phi }_{5}}{2.44*({\Phi }_{75}-{\Phi }_{25})}$$

where the abundance of particles with a certain *Φ* is here in area %; for instance, *Φ*_16_ is the size corresponding to 16 area % of the particle distribution^44,52^.

## Results

### XRPD

**Figure 4:**
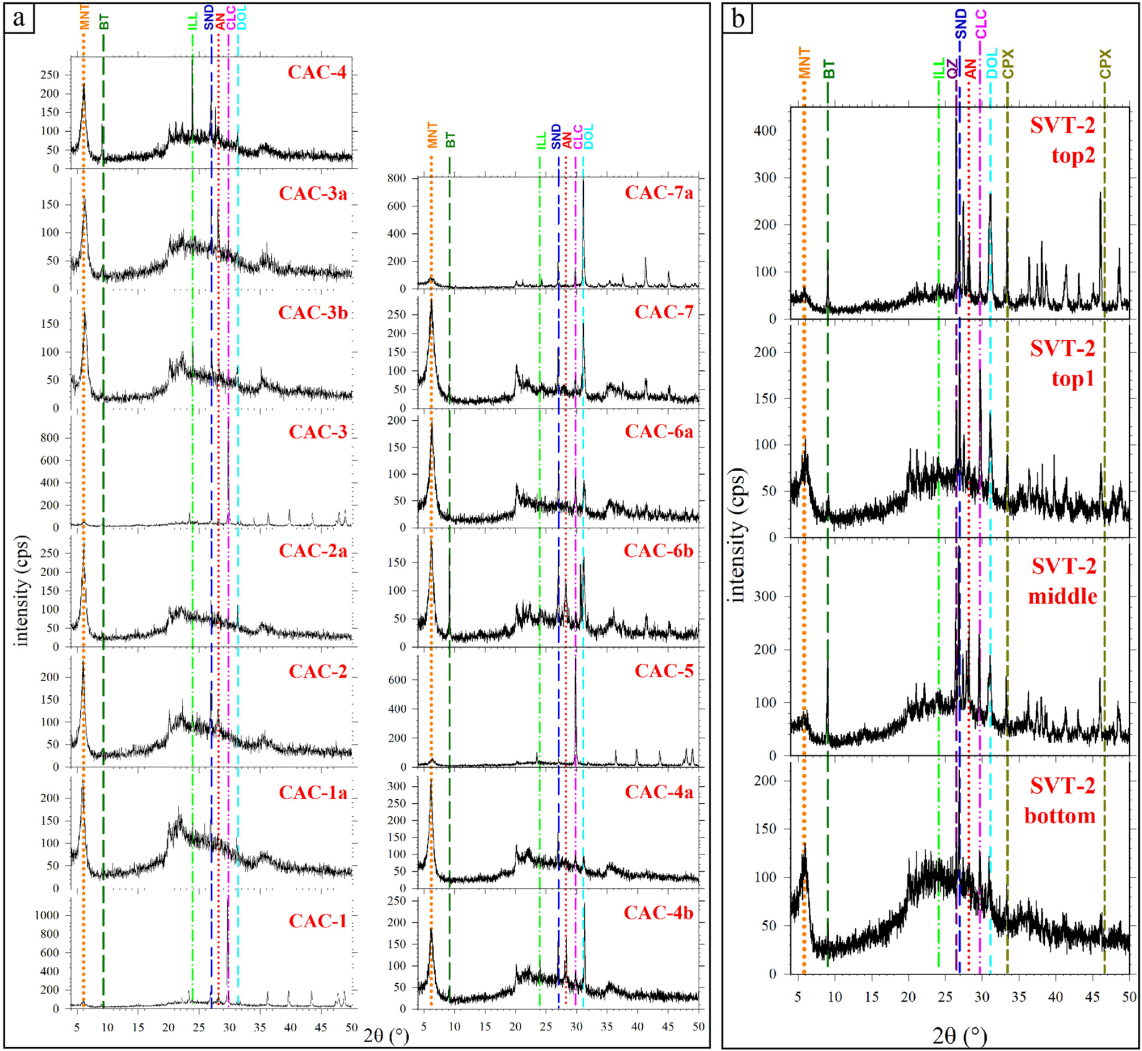
Stacked XRPD patterns as a function of vertical stratigraphy of the (**a**) CAC section (Castiglione a Casauria, Abruzzo) and (**b**) SVT-2 section (San Vittorino, Sant'Eufemia, Abruzzo). The coloured vertical lines correspond to crystalline standards from the ICSD database. These XRPD spectra are characterised by a large and relatively low-intense bulge around 18° and 32° of 2θ, indicative of the presence of a non-crystalline phase, except the samples CAC-1, CAC-3, CAC-5 and CAC-7a for whom the large shoulder is only barely detectable. Recognised crystalline phase are anorthite (AN), biotite (BT), calcite (CLC), clinopyroxene (CPX), dolomite (DOL), illite (ILL), montmorillonite (MNT), quartz (QZ) and sanidine (SND). The stratigraphic positions of these samples are reported in Fig. S1.

The four XRPD patterns of SVT-2 shown in Fig. 4b are stacked stratigraphically. The most prominent feature is the presence of a large shoulder with 2θ between about 18 to 33°. This broad hump decreases in intensity moving upwards along the section from the bottom (SVT-2-bottom) to top (SVT-2-top2) samples (Fig. 4b). The large shoulder is indicative of the presence of non-crystalline phase with silicate composition^40,42^. The identified faint Bragg peaks show that montmorillonite, illite and biotite sheet-silicates are present in all SVT-2 samples but with variable intensities (Fig. 4b). Quartz, sanidine and anorthite framework silicates are also invariably present, again with different intensities among samples, while the uniquely recognised Mg-Fe silicate is clinopyroxene (Fig. 4b). In addition to silicate minerals, calcite and dolomite are also present. The Bragg reflections are most intense when the amplitude of the shoulder between 18 and 33° of 2θ is low. At the bottom of the sequence (SVT-2-bottom), carbonate minerals have the lowest intensities, whereas the shoulder has the maximum intensity; this indicates that carbonates are present in small amounts and silicate glass is predominant. On the top of the section (SVT-2-top2), the situation is reversed (Fig. 4b). In the intermediate stratigraphy, the amount of silicate glass progressively decreases and that of carbonates increases (Fig. 4b).

A further, more detailed, XRPD analysis was carried out on the SVT-2-top2 sample. This uppermost portion of the section is characterized by a layer with rounded masses at the top (detached pseudo-nodules or rip-up clasts due to soft sediment deformation) composed by fine-grained ash surrounded by a relative coarser matrix (Fig. S3). The three sub-parts of this sample (Fig. S3) were analysed with XRPD, using a non-zero background sample-holder. All of them show the distinctive bulge of the amorphous phase with 2θ between 18° and 32°, overlapping that of the glassy sample-holder. Moreover, the detected minerals are the analogous to those present in the other SVT-2 samples (Figs. 4b, S3). The glass fraction of the sample appears to be more intense in the XRPD pattern labelled 3 and less in both patterns 1 and 2. The crystalline carbonate phases (calcite and dolomite), related to sedimentary material (authigenic cement), show more intense Bragg reflections in sample 2 (coarse matrix) and lesser pronounced in samples 1 (fine matrix) and 3 (shingle) (Fig. S3). Thereby, the top of the SVT-2 sequence is made of the same materials of the entire sequence, differing only in their aspects and possible grade of cementation (see below).

The fifteen XRPD CAC patterns shown in Fig. 4a are also stacked stratigraphically (Figs. 2a, S1). Apart from specimens CAC-1, CAC-3, CAC-5 and CAC-7a, the XRPD spectra have very similar features of those of SVT-2 (Fig. 4a). The former group, however, shows XRPD patterns with an almost flat background and high intensity Bragg reflections with hundreds and up to a thousand counts per second. These high intense Bragg reflections are due to carbonate minerals, while silicate minerals again show very faint to absent Bragg peaks (Fig. 4a). The other eleven XRPD patterns again show the large shoulder with 2θ between around 18 to 33°. The recognised minerals are again montmorillonite, illite, biotite, sanidine, anorthite, quartz, clinopyroxene, calcite and dolomite, with variable intensities (Fig. 4a). As with SVT, the CAC spectra show an inverse relation between amplitude of the large bump due to glassy phase compared with intensities of Bragg peaks, mainly those of carbonates (Fig. 4a).

### Mesoscopic features

The field and polished mesoscopic oriented samples allow discrimination of four different basic stratigraphic attributes: (i) unlayered or massive (structure-free), (ii) plane-parallel lamination, (iii) undulated-parallel lamination, (iv) complex, i.e. presence of a combined pattern of the other previous structures; the latter type also displays pseudo-layers and laminae intersecting each other and load, flame and fluid escape structures related to "inject/squeeze"^19^. In the field, the SVT-2 stratigraphic section is exposed on the top of a badland, where VRL-5.5 rocks are intensely fractured (Fig. 2b). The pervasive and intense fracture network partially obliterates the sedimentary features. From bottom to top there is a first plane-parallel horizon (type ii) also containing load cast structures (SVT-2-bottom), an undulated-plus plane-parallel layer (SVT-2-middle) (types ii and iii), then undulated-plus plane-parallel (type iii) plus an uppermost flame-bearing portion (SVT-2-top1) and finally a plane-parallel layered bed (type ii) including pseudo-nodules (SVT-2-top2) (Figs. 2b, S3). The basal contact with the underlying greyish claystone is very sharp. The overlying contact is with fossil-free siltites/claystones. This 80 cm thick interval is totally free of any structure indicative of mass transport or gravity flow layers and, even if stratified, internal layers are not separated by any other background hemipelagic sedimentation.

The mesoscopic texture and structure of the CAC column is poorly to moderately fractured and consists of a series of main beds, showing an overall thinning upwards trend (Figs. 2a, S1). The lowermost and massive horizon displays firstly a massive layer (CAC-1) (type i), followed by an undulated-parallel layer containing load plus fluid escape structures (CAC-1a) (type iii), then two further mesoscopic samples with complex load and dewatering structures (CAC-2, CAC-2a) (type iv). The successive horizon (CAC-3) shows plane-parallel laminations (type ii) then a new very complex (type iv) bed, including several soft-sediment deformation structures with convolute laminations and ash injections (CAC-3b). The uppermost sample shows a return to plane-parallel laminated facies (type ii) (CAC-3a) (Fig. 2a). The base of the deposit is juxtaposed on yellow calcareous sandstones with a sharp contact, while it gradually passes upwards to yellow–brown claystones on top. The mesoscopic appearance in the field suggests the existence of three similar main portions (Figs. 2a, S1).

### Microscopic features

The mesoscopic observations and XRPD outcomes need to be related and corroborated with microscopic observations. Vitroclasts can be subdivided by their sizes and morphology, such as: (i) blocky with curvy-planar surfaces and low vescicularity; (ii) vesicular with irregular shapes and smooth fluid-related surfaces; (iii) fine and irregular shaped consisting of several amalgamated globular masses; (iv) spherical or drop-like with smoothly curved surfaces, commonly attached and/or agglutinated; and (v) platy with curved surfaces, representing part of a bubble wall^11,49,53,54^.

The back-scattered SEM images of the four SVT-2 specimens clearly display very high amounts of a glass phase, a minor and variable presence of carbonate grains filling voids among silicate glasses and a very low content of silicate (magmatic and sedimentary) minerals (Fig. 3b). The glassy grains have sizes ranging from 224 µm (major axis) to the chosen threshold (see Methods: Image Analysis), with prevalent blocky (type i) and subordinated vesicular (type ii) morphologies (Figs. 3b, S4). A few glass grains contain bubbles with size ranging from 80 µm (major axis) down to the resolution threshold chosen (Figs. 3b, S4). Most grains have long/short ratios from 1:2 to 1:3, i.e. prismatic in shape, but a few clasts are extremely elongated with stretched bubbles (sample SVT-2-bottom in Figs. 3b, S4).

The seven lowermost CAC samples analysed with SEM micro-photographs display textural features very similar to SVT-2, being very rich in glass, poor in carbonate grains and with only minor silicate minerals (Figs. 3a, S4). Again, the sizes of these glassy grains vary from 198 µm down to the chosen resolution limit (see above), particles are also blocky (type i) in shape, but elongated particles with stretched bubbles (type ii) are slightly more abundant compared with SVT-2 (Figs. 3a, S4). The largest glassy and blocky grains are also fractured (Figs. 3a, S4).

**Figure 5:**
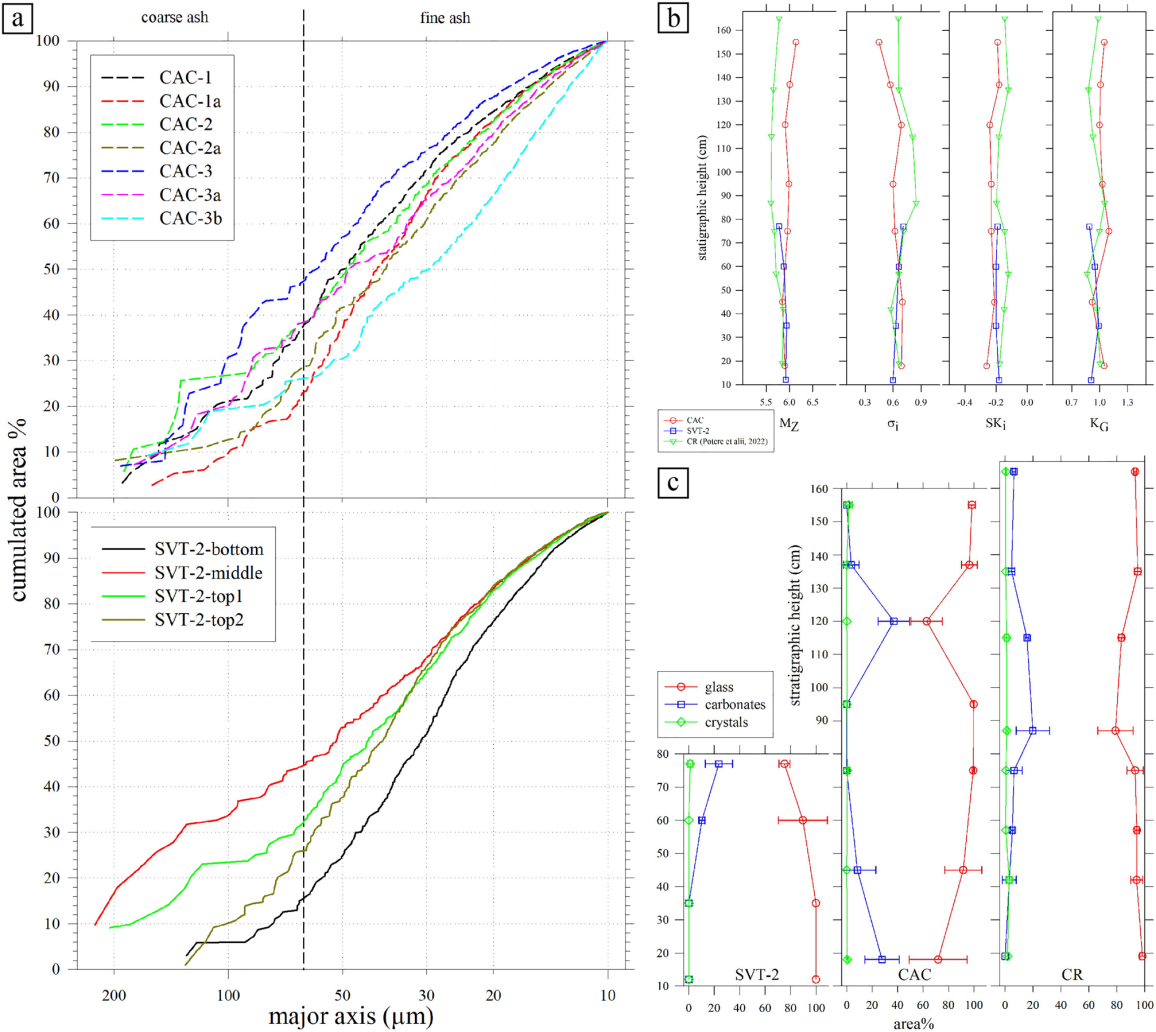
(**a**) Grain sizes *versus* area %. The major axis corresponds to the major axes (µm) of equal-area ellipses quantified by image analysis on BS-SEM microphotographs (Fig. 3) at 200×; (**b**) vertical variations of the grain-size distribution parameters (see Table S4); (**c**) abundance of glasses, carbonates and crystal phases, calculated by image analysis on 3 representative BS-SEM microphotographs per thin section at a magnification of 100×; the vertical size of diagrams scale with the stratigraphic height in the field (Fig. 2). The data labelled CR refer to the Camporotondo section (Potere et al. 2022, see Fig. 1).

The slight lithification of both the SVT-2 and CAC sections prevents 3D grain-size determinations with classical sieve and flotation methods. Grain-size distributions of the samples were determined by image analysis on thin sections^44,45,51^. The 2D grain size distribution curves are here represented by the relationship between major axis of volcanic glassy grains *versus* their (cumulated) area % (Fig. 5a). Glassy clasts are invariably in the ash range (< 2 mm), with maximum lengths around 200 μm, with dominance of very fine ash grains with respect to coarse ones (Fig. 5a).

The same linear dimension and abundance in area % of any 2D glassy grains were used to determine the classical whole granulometric parameters^52^, mirroring those obtainable by 3D determinations^44,45,51^ (Table S4, Fig. 5b). In line with the distribution of major axis (Fig. 5a), the mean size *M*_Z_ changes only slightly between samples, being between 5.78 and 6.14 Φ. Specifically, the mean grain size in SVT-2 has an average value of 5.88 Φ and tends to slightly decreases upwards, while in CAC the average value is 5.96 Φ with an irregular and limited trend that faintly increases upwards (Table S4, Fig. 5b). In a similar way, the other three grain size parameters show only limited variation among all the eleven analyzed samples.

The qualitative amounts of carbonate (sedimentary) fractions observed in thin sections and detected by XRPD vary from completely absent to abundant, whereas silicate (magmatic and sedimentary) contents are always very small (Figs. 3, 4, S4). A quantitative reappraisal is provided by image analysis on BS-SEM microphotographs (Fig. 5c). Such microscopic determinations provide the actual abundance of carbonates and pyroclasts in area %, to fully constrain the sedimentary and volcanic fractions^21,45,55^.

Along the SVT-2 section, crystals are invariably < 2 area %, while carbonates range from almost 0 to around 25 area % moving upward, in line with the XRPD results (Figs. 2b, 4b, 5c). For the CAC unit, the quantity of silicate crystals is again very low (< 2 area %). Carbonate contents are variable, first decreasing from about 25 area % in CAC-1 to 0 in CAC-2 and CAC-2a, then increasing to about 40 area % in CAC-3 and finally decreasing again to about 0 in CAC-3b (Figs. 2a, 5c). The abundant carbonate in the two specimens in the CAC section is qualitatively corroborated by their XRPD patterns, i.e. the intensities of Bragg reflections *vs* amorphous bulge (Fig. 4a). Based on this correspondence, it is expected that the CAC-5 and CAC-7a samples are also rich in carbonates (Figs. 4a, S1). Unlike the SVT-2 unit, the CAC section hosts several horizons extremely rich in carbonates, starting from its base; the distance from these horizons decreases moving upward in agreement with mesoscopic observations (Figs. 3, S1). The carbonate phases are either in the form of cement filling spaces intra- and inter-shards (like in CAC-3), whereas carbonate clasts mixed with pyroclastic materials (like in SVT-2-top1 and SVT-2-top2) are extremely limited (Figs. 3, S4). The amount of vesicles is not accurately measurable due to the large quantity of unconfined bubbles (Figs. 3, 4, S4).

### Whole-rock chemical compositions

The major oxides and trace elements of the two SVT-2 and two CAC samples richest in volcanic materials and poorest in sedimentary carbonates are reported in Tables S3 and S5. On the whole, the quantities of major oxides show little change among the four samples with the maximum differences shown by SiO_2_, CaO, CO_2_ and H_2_O. The variation of H_2_O is related to H_2_O^−^, which changes between 1.5 to 4.5 wt.%, whereas H_2_O^+^ shows very little difference ranging from 6.8 down to 6.2 wt.% in the four samples (Table S3). The SiO_2_ amounts are inversely correlated with those of CaO and CO_2_, but unrelated to those of H_2_O^−^ and H_2_O^+^, whereas the amounts of LOI and of those of CO_2_ + H_2_O^+^ + S^tot^ are similar in all the samples (Table S3, Fig. S5). Finally, the FeO/Fe_2_O_3_ ratio is high for the top layer of SVT-2, whereas is close to unity for the SVT-2-bottom and both the CAC samples (Table S3).

### Micro-chemical compositions of glassy grains

**Figure 6:**
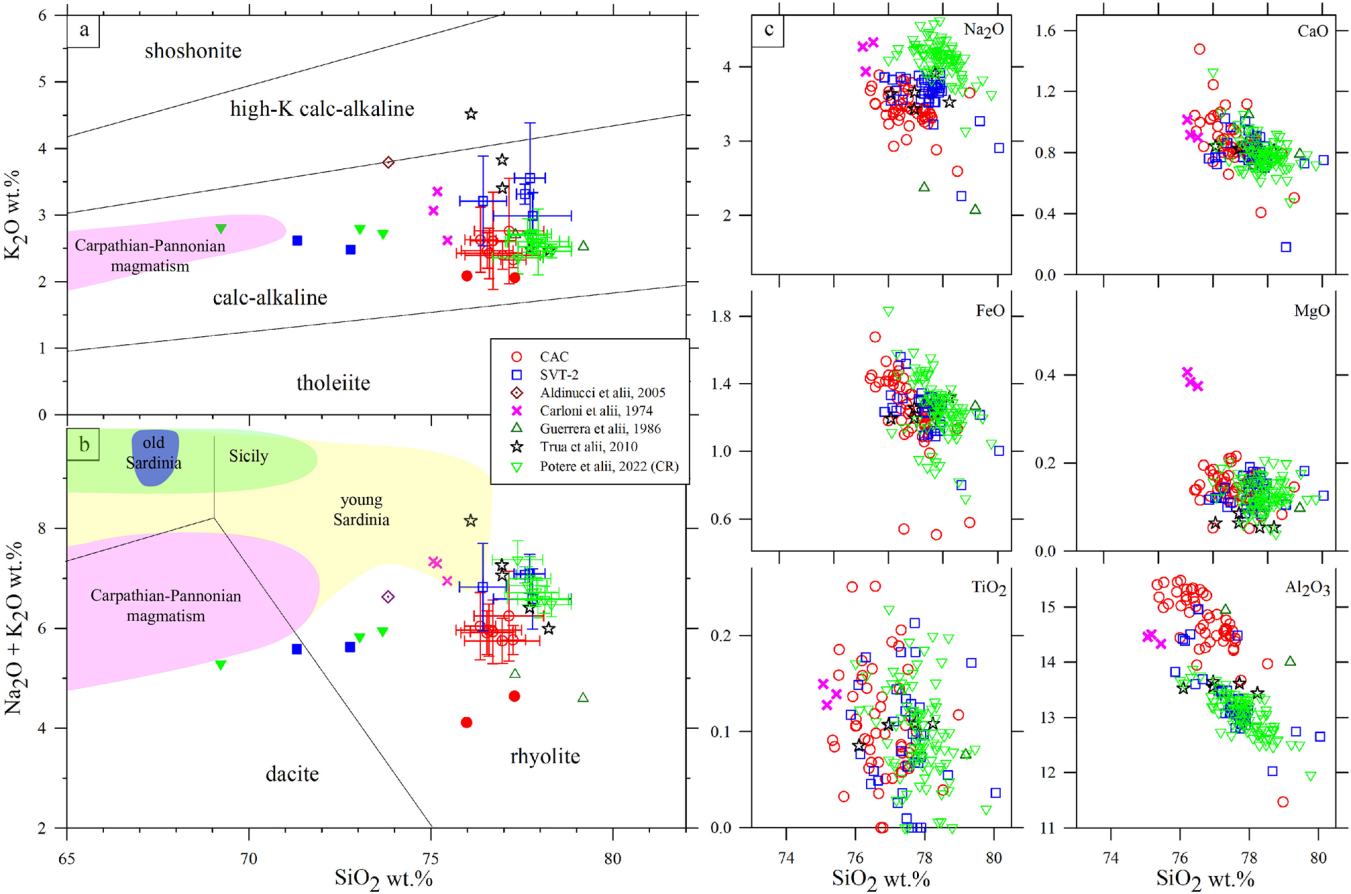
(**a**) SiO_2_ vs K_2_O (Peccerillo and Taylor 1976), (**b**) TAS diagram (Le Maitre et al. 2002) and (**c**) Harker diagrams of the VRL-5.5 glasses (empty symbols) measured with EPMA and bulk (filled symbols) analyses values (on a dry basis from the data in Tables S3, S6). Old Sardinia, Mio-Pliocene stage 11.8–4.4 Ma (Peccerillo 2017); Sicily 7 Ma to present (Peccerillo, 2017); Carpathian-Pannonian magmatism 6–2.5 Ma calc-alkaline rocks (Harangi and Lenkey 2007); young Sardinia, Plio-Quaternary stage, 3.9–0.1 Ma (Peccerillo 2017). All the data from this and previous studies are clustered in limited compositional domains, except few samples. The data of Potere et al. 2022 refer to the Camporotondo section (Marche region, see Fig. 1).

The contents of major oxides of glassy grains are reported in Table S6, along with previous and similar determinations performed on other lateral-equivalent VRL-5.5 samples (see below). The amount of major oxides determined with EPMA differs little from those of bulk chemistries (Tables S3, S6), in agreement with the low amount of crystals detected by XRPD and especially by textural outcomes (Figs. 3, 4, 5c, S3). The most important difference between EPMA and bulk geochemical data is related to SiO_2_. Silica is by far the most abundant oxide in glassy clasts and is also the most abundant crystal-chemical component in sedimentary sheet-silicate minerals, like montmorillonite and illite (Figs. 4, S3). Nonetheless, these minerals have a relative low content of SiO_2_ (< 50 wt.%). Hence, the slight lower amounts of SiO_2_ in bulk samples with respect to glass clasts can be mainly attributed to the presence of sheet silicates. In the limit of the high LOI contents SVT-2 and CAC glasses have similar rhyolitic compositions in the TAS (Total Alkali vs Silica) and SiO_2_ *vs* K_2_O diagrams (Fig. 6a, b); the variations of the other major oxides with respect to SiO_2_ are displayed in Fig. 10c. All these geochemical determinations overlap with those determined in previous investigations (Fig. 6) on lateral stratigraphically equivalent outcrops and fall in restricted compositional fields.

## Discussion

### Magmatic features of VRL-5.5 sites

The two SVT-2 and CAC sections analysed here share a significant overlap in the major oxides composition of glasses with previous investigations (Fig. 6), as well as the same stratigraphic position (Fig. 1c) and mesoscopic and microscopic features (textures of pyroclasts)^24–32,56^ (Figs. 2, S1). These similarities reveal (see below) that SVT-2 and CAC sections belong to the same volcanic eruption and magmatic process that occurred at 5.5 Ma^33^. Thereby, the SVT-2 and CAC investigated sections are the two new and southernmost occurrences of the same VRL-5.5 volcanic explosive event (Fig. 1a, b). Quartz, plagioclase, alkali-feldspar, biotite and clinopyroxene are the crystalline phases (Figs. 4, S3) related to the magma that generated this volcanic material^24,25,32^. Moreover, the content of these minerals in SVT-2 and CAC is extremely low in line with previous investigations on the VRL-5.5 (Figs. 3, 5c, S4); they occur exclusively like tiny crystals (microlites and micro-phenocrysts) into the glassy clasts and practically never as single loose minerals (Figs. 3, S4). They can be thus considered related to magmatic solidification and are not attributable to sedimentary processes. In parallel, the rarity of single and loose minerals suggests a distal provenance of these volcanic materials or alternatively a very high aphyric character^2,3,54^ (see below).

**Figure 7:**
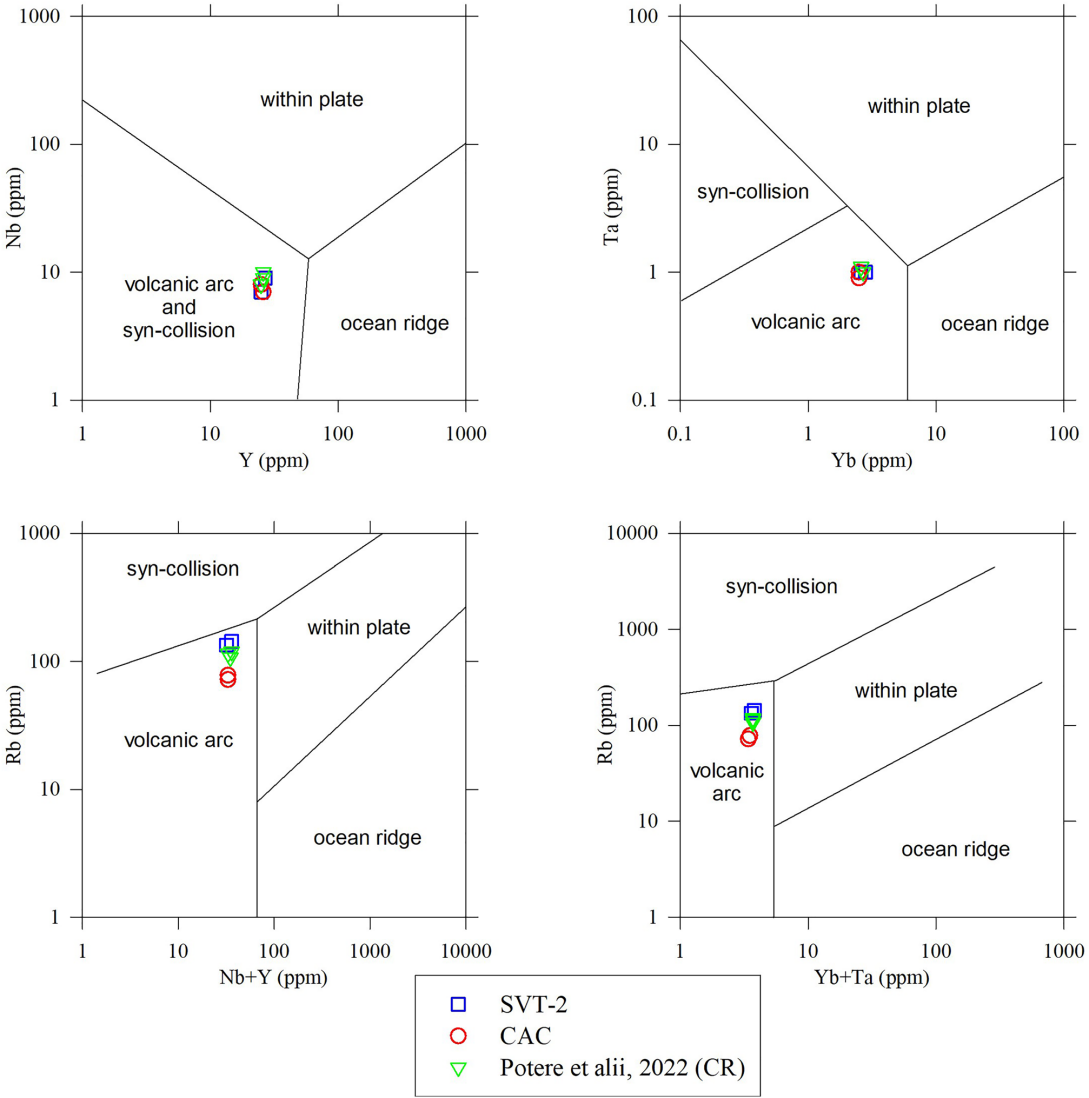
Immobile trace elements proxies for tectonic interpretation of high-silica-content rocks (modified from Pearce et al. 1984) (from data in Table S5). All axes are logarithmic. The data labelled CR refer to the Camporotondo section in Potere et al. 2022, see Fig. 1).

The amount of dissolved H_2_O in glass was not directly determinable with classical FTIR measurements. In turn, we are forced to indirectly infer this content by comparing the difference between 100 and sum of oxides wt.% in EPMA analyses (Table S6) with H_2_O^+^ bulk determinations (Table S3) as displayed in Fig. S6. Since these differences are similar in the four analysed samples of this study (two SVT-2 and two CAC) and from the section of Camporotondo (see Fig. 1a)^57^ it can be thus concluded that the amount of dissolved H_2_O in the VRL-5.5 is between 5 and 9 wt.% (Fig. S6). These high H_2_O contents can be due to primary magmatic water and to secondary and post-deposition hydration by seawater^58^. However, in absence of H_2_O profiles and/or its isotopic signatures (H and/or O) we cannot quantify the fraction of magmatic *vs* submarine water in these glasses. The actual determinations of the primary magmatic water content will further clarify also the type of petrological processes that produce such eruptions. However, the magmatic signature of the VRL-5.5 can be here directly inferred using the bulk immobile elements^59,60^, as displayed in Fig. 7. All the minor and trace immobile elements analysed here and from the Camporotondo section converge on the field of volcanic arc and syn-collisional domains (Fig. 7).

### Provenance and magnitude of the VRL-5.5 eruption

The unique previous hypothesis on the provenance of the VRL-5.5 magma suggested a paleo-Ponza source linked to the opening of the Tyrrhenian basin^32^. Such hypothesis is improbable, since immobile elements suggest a convergent setting (Fig. 7) that is contrary to the extensional features linked to the Tyrrhenian domain. The possible provenance of the VRL-5.5 pyroclasts from a Central or Southern Tyrrhenian source is also poorly supported by the location of outcrops and related thicknesses reported in Fig. 1. A provenance of a pyroclastic cloud from the paleo-Tyrrhenian area expanding towards NW should accumulate significant deposits also in Tuscany, where the VRL-5.5 is reported in the SQ site but with only 1 cm of thickness (Fig. 1a). Similarly, under a prevalent expansion of the explosive pyroclastic cloud from the (paleo) Central or Southern Tyrrhenian source towards W, the two SVT and CAC sections should be the thickest ones with respect to all the other VRL-5.5 sites (Fig. 1a); moreover, other sites in the southern Italy should expose the VRL-5.5.

**Figure 8:**
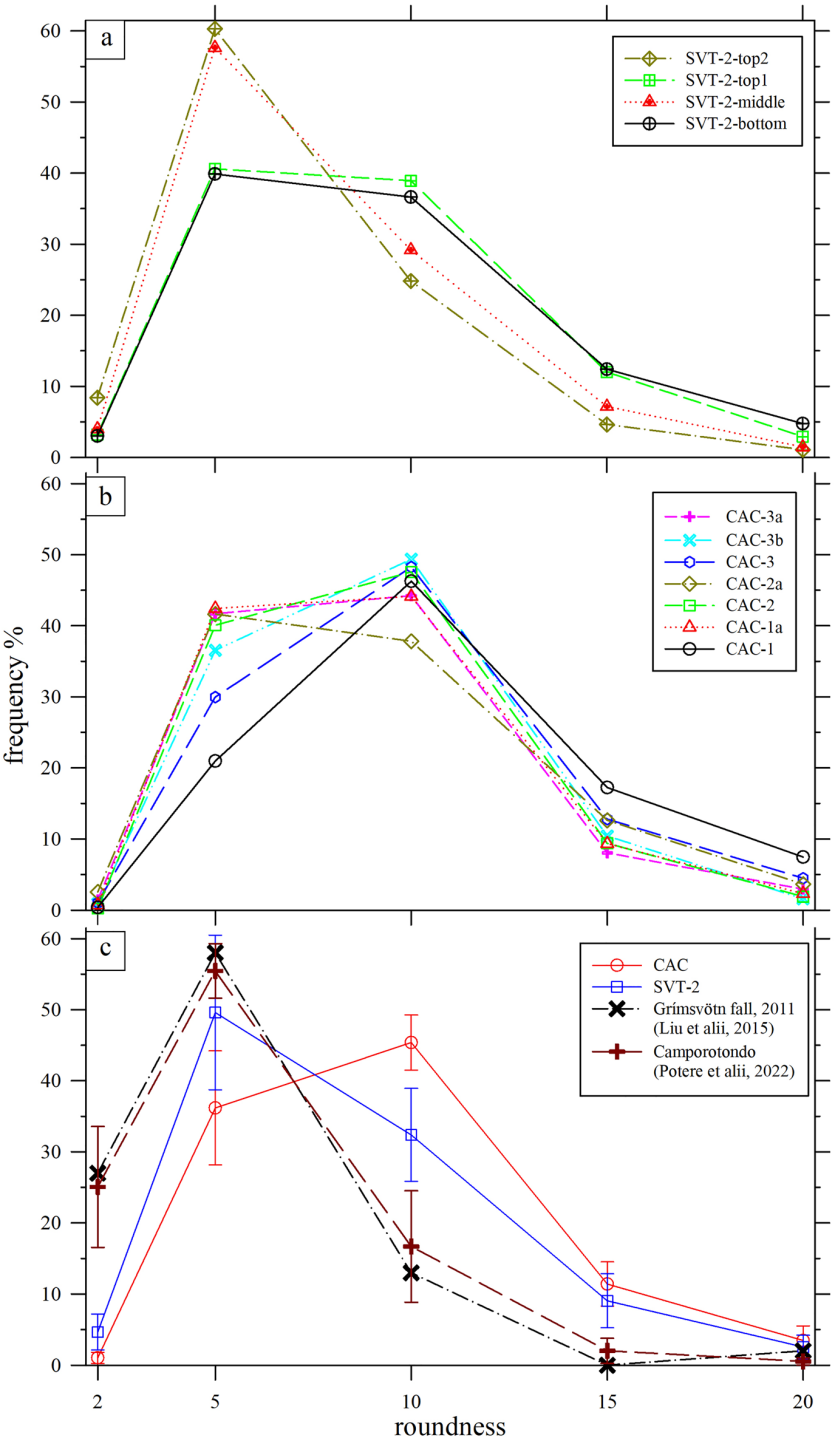
Roundness frequency of all equal-area ellipses of glass shards of (**a**) SVT-2, (**b**) CAC, quantified by image analysis on BS-SEM images with magnification of ×200. 11 classes are considered, to cover all experimental data, from < 2 to 45–50. Only classes < 20 are reported, being representative of the material; (**c**) the Grímsvötn-2011 fall (sample G6, 60 km from the vent, Liu et al. 2015) and Camporotondo (CR, Potere et al. 2022) data are reported for comparison with CAC and SVT-2 mean values plus standard deviations.

This picture is at the moment limited by the available grain-size data of many VRL-5.5 sites. In fact, only the SVT-2 and CAC sections analysed here and the Camporotondo one^57^ have available quantitative textural data (Figs. 5, 8, S7), while the other VRL-5.5 sites (Fig. 1a, b) must be still quantified. Their very low amount of crystals (Fig. 8), especially the loose ones, can be indicative of: (i) an (improbable) mineral-free magmatic eruption, or (ii) more probably a classical distal explosive fallout deposit^2,3,54,61–63^. This second option is perfectly in line with the very homogeneous, extremely fine (*M*_Z_ ~ 6), well sorted (*σ*_i_ ~ 0.6) and practical identical grain-size distributions of the VRL-5.5 in SVT-2 and CAC sections, as well as in the Camporotondo outcrop (Figs. 5a, b). In detail, it is the Camporotondo section that displays the slight larger grains with respect the SVT and CAC ones (Fig. 5b). It can be thus speculated that the Camporotondo outcrop could be located closer than both SVT and CAC to the source of the VRL-5.5 pyroclasts.

The thickness of the primary tephra fallout, both proximal and distal, is useful in the reconstruction of some parameters of the eruption^64^. Unfortunately, except SQ, the available VRL-5.5 outcrops are all aligned in the peri-Adriatic basin along roughly N-S trending exposures (Fig. 1a) and proximal deposits are lacking or still unknown. Here, we can only consider an original thickness on the order of tens of cm of the VRL-5.5 fallout as SVT-2. Thicknesses of several tens of cm even at 50–100 km from their source are indeed indicative of large magnitude eruptions^8,22^, with M > 7 and VEI in the same magnitude range^65^. In this context, an insightful comparison can be made with grain-size, thickness and distance values reported for several large and well-known eruptions (Pinatubo 1991, M 6.1, Los Chocoyos 84 ka, M 8.2, Toba 74 ka, M 8.8, Campanian 39 ka, M 7.7)^3^. At 1000 km from its vent, the Campanian Ignimbrite has thicknesses of ~ 80 cm; moreover, at 1000–2000 km from their emission sites, only very large eruptions like those of Toba and Campanian Ignimbrite have grain sizes on the order of *M*_Z_ of 6, in line with VRL-5.5 (Fig. 5b), equivalent to few tens of μm. Indeed, further textural and depositional (see below) constraints on other sites (Fig. 1a) are required to extend the real extension of the VRL-5.5 eruption. Nonetheless, it can be speculated that the tens of cm of thicknesses of the VRL-5.5 outcrops (Fig. 1a), assuming that all are entirely primary (see below), are more plausibly related to some unknown, buried and large volcanic structure active at 5.5 Ma.

We have already argued that a western or southern-western source of the VRL-5.5 is improbable, and this is further supported by geochemical comparisons between the VRL-5.5 rocks and those emitted around 5.5 Ma (Fig. 6a, b). In the European terrains the active magmatic sources at that time were those from old Sardinia, Sicily, the Carpathian-Pannonian area, and young Sardinia, spanning from 11.8–4.4 Ma, 7–0 Ma, 6–2.5 Ma and 3.9–0.1 Ma, respectively^66,67^. It must be considered that the actual bulk chemical composition of the VRL-5.5 could differ from those reported here in Fig. 6a, b and Table S3 due to the possible lacking of some minerals (see before) and addition of non-magmatic water. Under these considerations, the Carpathian-Pannonian magmas are those closest to the VRL-5.5 in terms of geochemistry and age (Fig. 6a, b); these magmas are also related to the convergent tectonic processes^67^, so in line with the scenario suggested by the VRL-5.5 immobile elements (Fig. 7). In summary, the VRL-5.5. sites in the Apennines are believed to represent the eastern and distal deposit of a large eruption occurred in the Carpathian-Pannonian arc at 5.5 Ma. This suggestion must be proven with similar analyses on the other known VRL-5.5 outcrops (Fig. 1a) and at lesser distance with the Carpathian-Pannonian area.

### Deposition of the VRL-5.5 pyroclasts

The VRL-5.5 is not a canonical tephra due to its lithification^2,3,54^. The hypothesis of a primary fallout in air, followed by a sinking in seawater was already proposed^30^, but poorly confirmed by many other studies. In fact, this deposit has been more commonly ascribed to a very large gravity current^27,28,32^. Indeed, all these previous studies base on mesoscopic observations, whilst microscopic characterisations are not available. The field plus 2D mesoscopic and microscopic features (Figs. 2, 3, S1, S4) allow reconstruction of the SVT-2 and CAC VRL-5.5 complex depositional processes. The carbonate minerals mainly infill voids among volcanic shards (Figs. 3, S4). Therefore, after the deposition of glasses they directly precipitated by circulating seawater rich in dissolved carbonates^16,18^. The presence of montmorillonite and illite cannot be related to any magmatic processes and are thus formed in the sedimentary Messinian submarine setting or after their exhumation at the expense of glass particles^10,20,68,69^. These two sheet-silicates phases detected by XRPD (Figs. 4, S3) are unobserved by SEM (Figs. 3, S4), indicating that their low content coupled with their extremely low sizes, makes them observable only at a sub-micrometric scale. It can be thus concluded that carbonates, montmorillonite and illite are related to chemical sedimentary processes occurred after the deposition of VRL-5.5 tephra (before its lithification); biotite, clinopyroxene, feldspars, quartz and silicate glasses are exclusively linked to magmatic phases (Figs. 3, 4, S3, S4).

The content of sedimentary carbonates is variable from 0 to around 40 area % along the two stratigraphic sections (Fig. 5c). A similar situation was observed in the Camporotondo section^57^. For the SVT-2 section, the carbonate fraction increases upward, while for the CAC and Camporotondo sections it both increases and decreases (Figs. 2, 4, 5c, S1). These differences between SVT-2 and CAC (and Camporotondo) sections are in line with field observations, and can be explained in light of their different thickness despite their proximity (Figs. 1a, 2, 5c, S1), i.e. the SVT-2 was deposited by a unique event, whereas CAC by at least two events. In detail, all the four SVT-2 samples and seven CAC samples display very similar grain size features (Figs. 5a, b, 8, S7, Table S4), although they show mesoscopic and field differences (Figs. 2, S1). The grain size distributions show a purely ash-sized material (Figs. 5a, S7), characteristic of a distal air-fall deposit^61–63^, displaying no trend as a function of the thicknesses of the two sections (Figs. 5b, c). In the intrinsic limitations of these 2D measurements, the two SVT-2 and CAC sections have practically identical size dimensions of glassy grains, with very limited or no variation as a function of their internal stratigraphy (Figs. 5b, c).

A further refinement of the grain size features of the volcanic glass fractions is provided by their distributions of major *vs* minor axes, aspect ratio and orientation in 2D of the major axis of equal-area ellipses, which strongly overlap among themselves (Fig. S7). The aspect ratios of glass shards are equals or lower than 4, i.e. the glass shards are equant to poorly prismatic (Fig. S7). The polar graphs of the orientation of the major axis of the glassy particles are all around the theoretical mean value of 8.3% per class (a perfect random distribution), testifying to the absence or minor presence (SVT-2-top1, CAC-3 and CAC-3a) of a fabric (Fig. S7). This is attributable to the low anisotropy of 2D glassy ash clasts (see above) and to low or absent depositional effects, e.g. absence of primary mass transport.

The 2D glassy particles were also further characterized by their roundness. Starting from a certain primary clast shape, the decreasing of its roundness towards 1 (sphere) implies a proportional augmentation of mechanical abrasion and transportation [modified from^49,70,71^]. Here, in the absence of roundness measurements at different distance from a common source or volcanic vent, it is possible to compare such parameters only along and between each section. All the measured ash clasts have roundness > 2 and < 15 (Fig. 8), show limited difference among themselves, and are similar to the recent primary deposited tephra fallout from the Grímsvötton volcano^49^, reported for comparison. The Camporotondo section is practically identical to those of SVT and CAC (Fig. 8). Hence, the SVT-2 and CAC ash shards have roundness features similar or even less rounded than a pristine historical primary tephra fallout sampled at > 60 km from its vent (Fig. 8).

To identify the depositional processes that emplaced these two VRL-5.5 rocks, we have to consider the absence of: (i) typical sorting and sedimentary structures of turbulent turbiditic/gravity deposits, (ii) fossils and carbonate clasts and (iii) intercalated hemipelagic horizon(s) inside the VRL-5.5 (Figs. 2, S1), coupled with the presence of: (i) very fine and homogeneous grain sizes of glasses (Figs. 5, S7), (ii) the anisotropic shape of glass clasts and (iii) their low roundness (Figs. 8, S7). Therefore, the VRL-5.5 results mainly from a continuous sinking of a distal fallout around 5.5 Ma ago in seawater, for both SVT-2 and the lowermost horizon of the CAC submarine sites. In other words, the hypothesis of a primary fallout deposited in seawater is the most plausible, in line with Aldinucci et alii 2005, whereas a VRL-5.5 genesis from a giant gravitative process is improbable^27,28,32^.

The significantly greater thickness of the CAC (220 cm) deposit than SVT (80 cm) and the presence of mesoscopic sedimentary structures (Table S1, Fig. 2), such as parallel and undulated plus convolute laminations, slumped pseudo-beds and fluid escape structures (Fig. 2), could not exclude the occurrence of minor, local, limited and gentle post-depositional reworking of the primary pyroclastic materials. At the same time, the most part of these two VRLs are attributable only to direct sinking of pyroclasts in seawater. This holds for the uppermost portion of the CAC section that probably represents a local redeposition (Fig. S2). Similarly, the distribution of the same VRL-5.5 deposit is variable in the Marche outcrops described in the literature, such as: 150 cm at Colle Gallo and Amandola, 200 cm at Camporotondo, 120 cm at Maccarone and 80 cm at Calcinelli^24,25,32^, while the thicknesses reported in the literature for the northernmost outcrops are 40 cm for Campea and Piavola (Fig. 1a)^32^. This variation in thickness further hints at local remobilizations of the other outcrops of the VRL at 5.5 by very low-energy and local mass currents from still unconsolidated and water-logged VRL-5.5 pyroclastic deposited from nearby places. These remobilizations occurred during or immediately after their deposition in the post-evaporitic basin with relative steep flanks.

This new general interpretation suggests reconsideration on the provenance and deposition mechanism of all the VRL-5.5 sections is required (Fig. 1). Further sections need be studied in detail following the analytical protocol reported here to adequately bracket the deposition of this probably large explosive eruption. The field, mesoscopic and microscopic analytical protocols used here are valuable to reconstruct the depositional history of other ancient and lithified volcanic-rich rocks and horizons.

## Supplementary Information

Supplementary Information 1.

Supplementary Information 2.

Supplementary Information 3.

Supplementary Information 4.

Supplementary Information 5.

Supplementary Information 6.

Supplementary Information 7.

Supplementary Information 8.

Supplementary Information 9.

## Data Availability

All essential data generated or analysed during this study are included in this published article (and its supplementary information files). Other data are available from the corresponding author on reasonable request.
