# Supplementary figures and images for: Provenance and deposition of a lithified volcanic-rich layer (VRL-5.5) at 5.5 Ma from Central Apennines (Italy)
Source: Sci Rep. 2023 Apr 27;13:6880. doi: 10.1038/s41598-023-33256-2 (PMC10140287; doi:10.1038/s41598-023-33256-2)

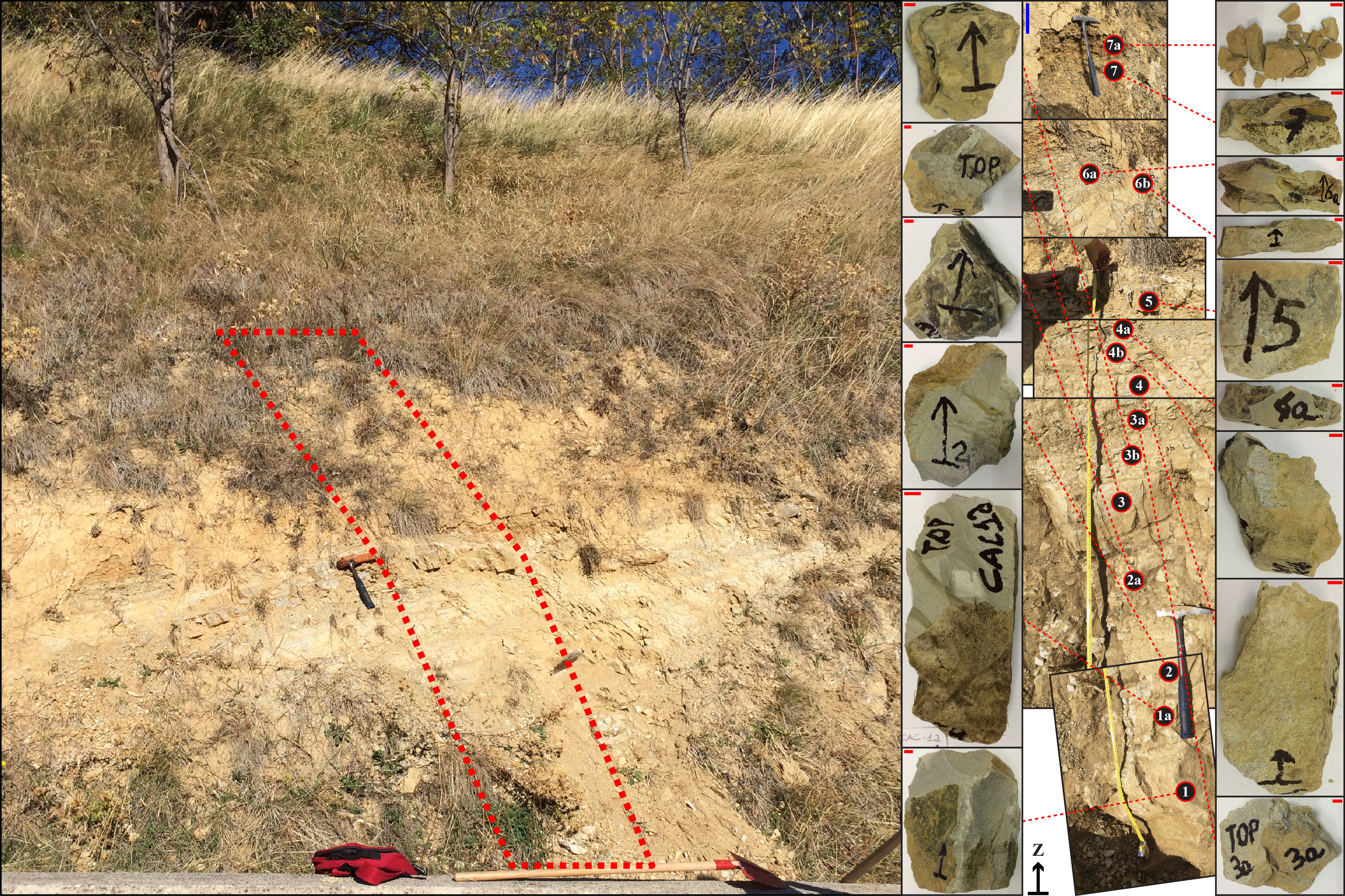

Supplement: Supplementary file 1 — Supplementary Information 1. [file 41598_2023_33256_MOESM1_ESM.jpg]

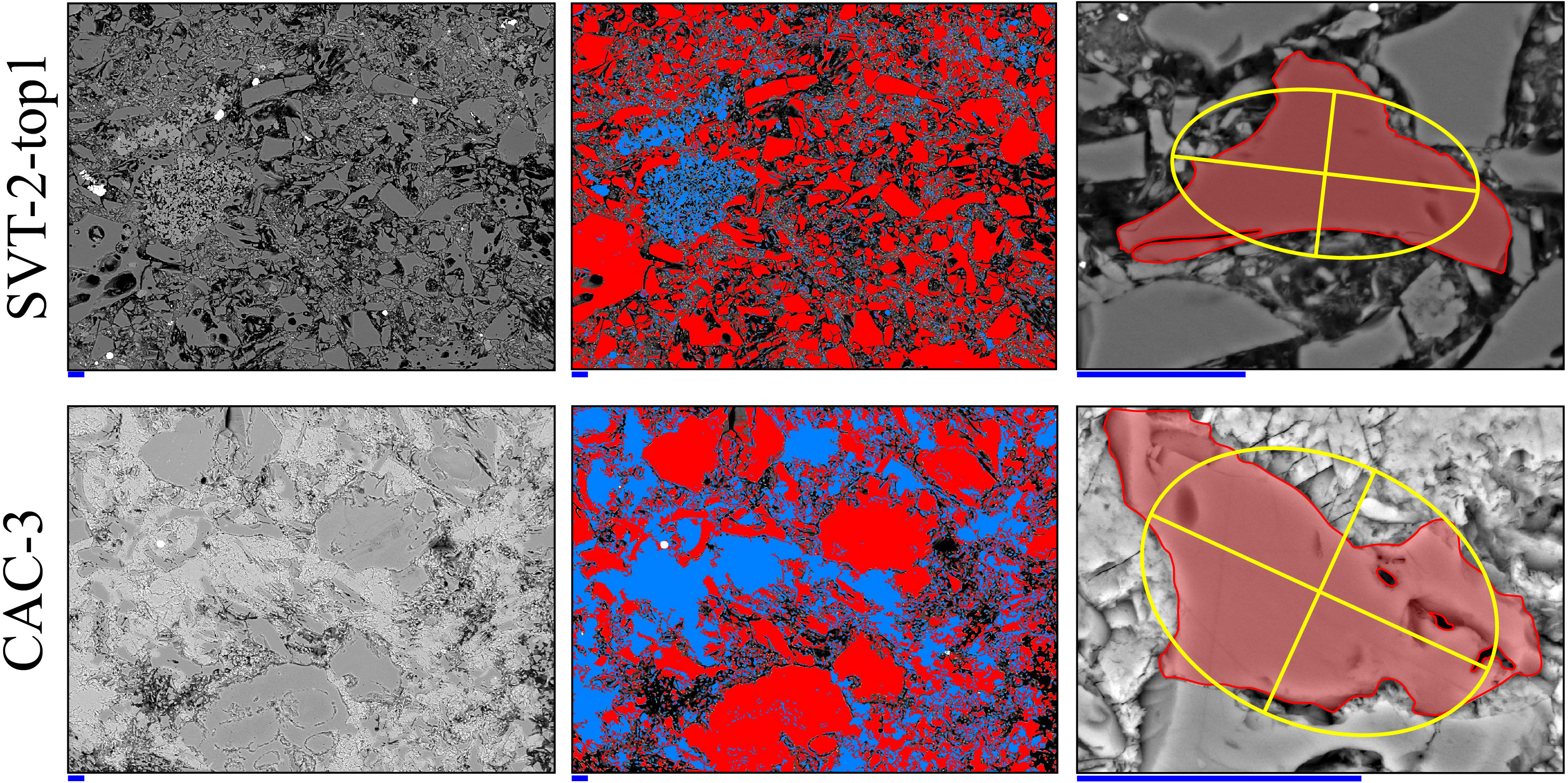

Supplement: Supplementary file 2 — Supplementary Information 2. [file 41598_2023_33256_MOESM2_ESM.jpg]

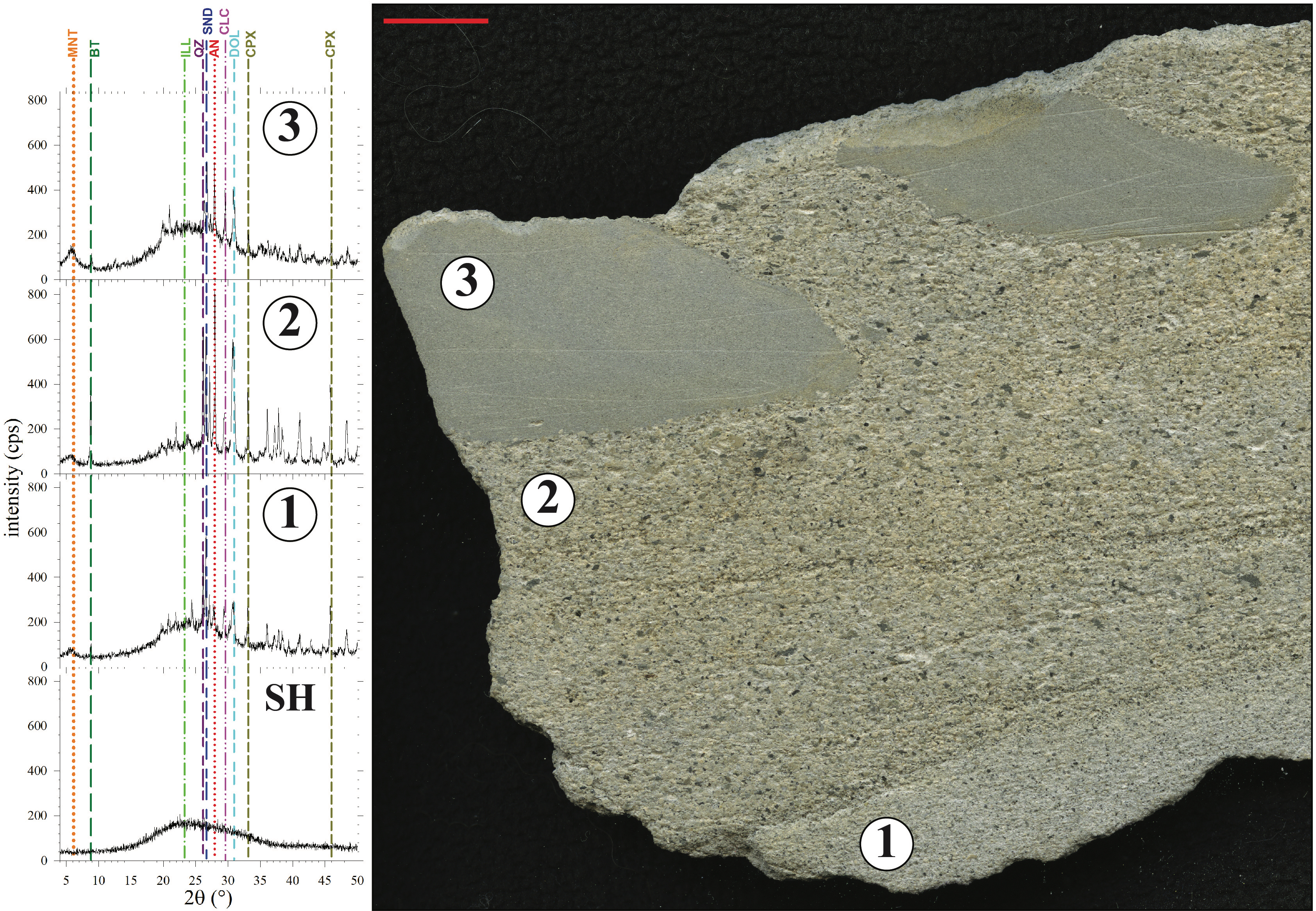

Supplement: Supplementary file 3 — Supplementary Information 3. [file 41598_2023_33256_MOESM3_ESM.jpg]

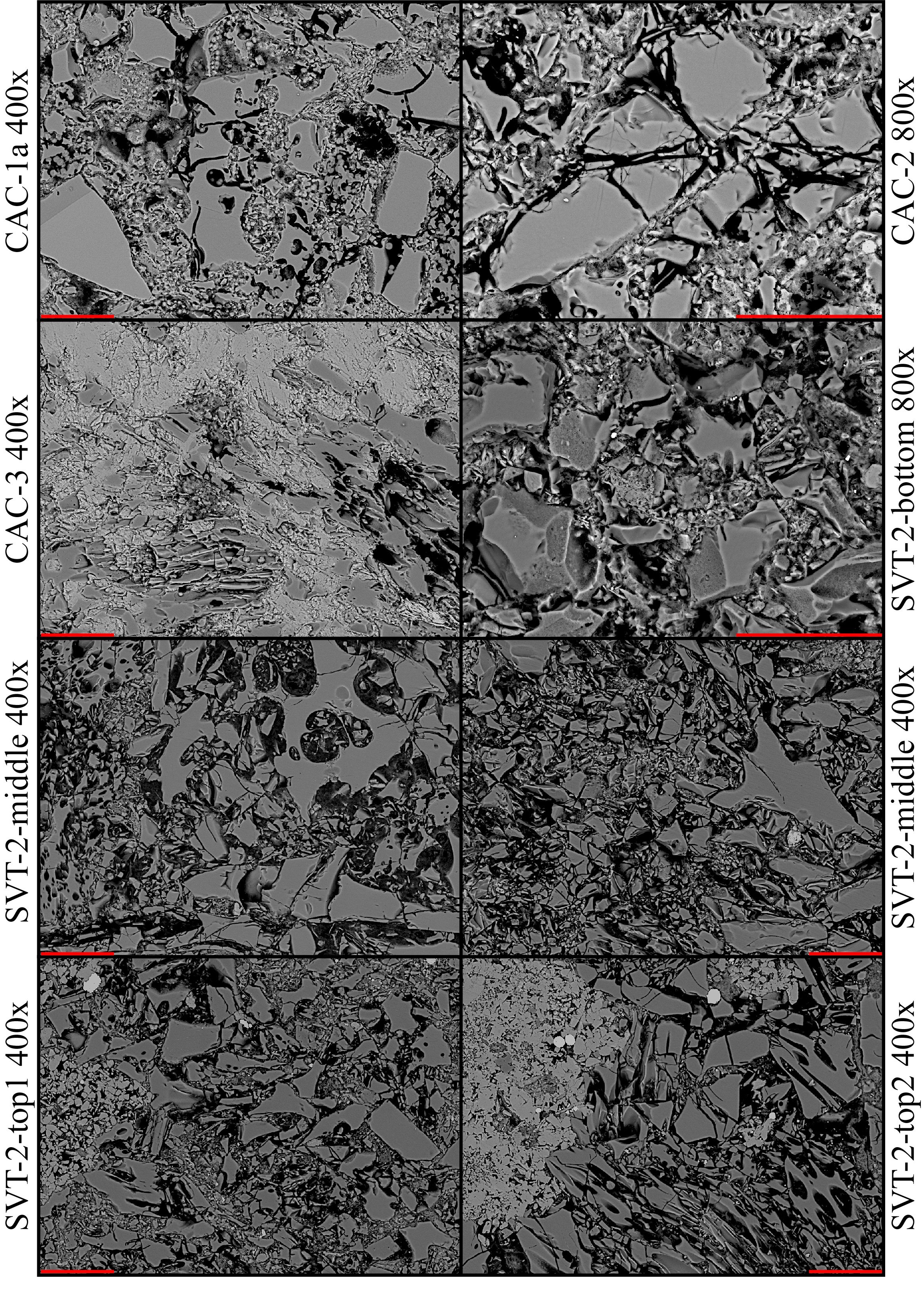

Supplement: Supplementary file 4 — Supplementary Information 4. [file 41598_2023_33256_MOESM4_ESM.jpg]

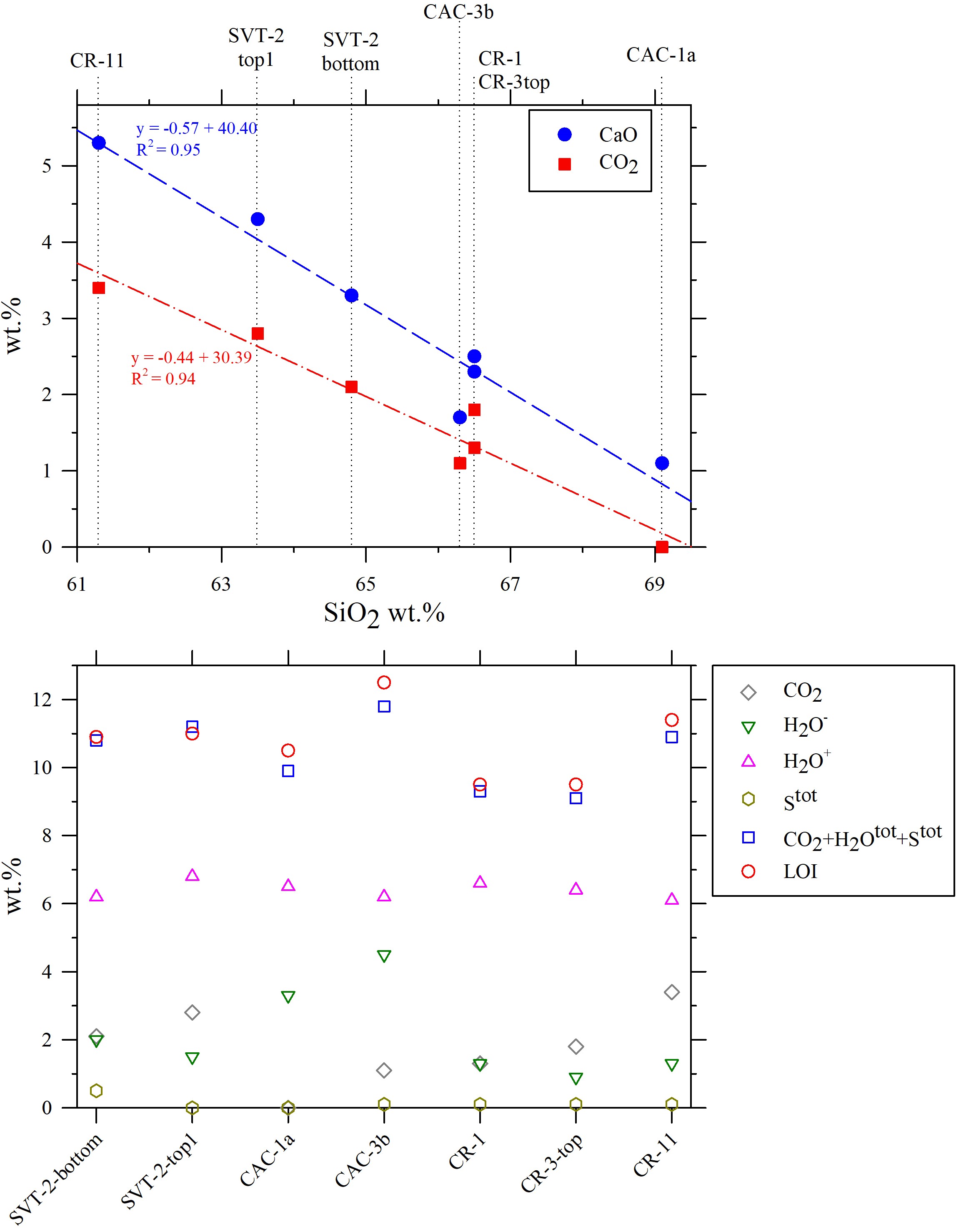

Supplement: Supplementary file 5 — Supplementary Information 5. [file 41598_2023_33256_MOESM5_ESM.jpg]

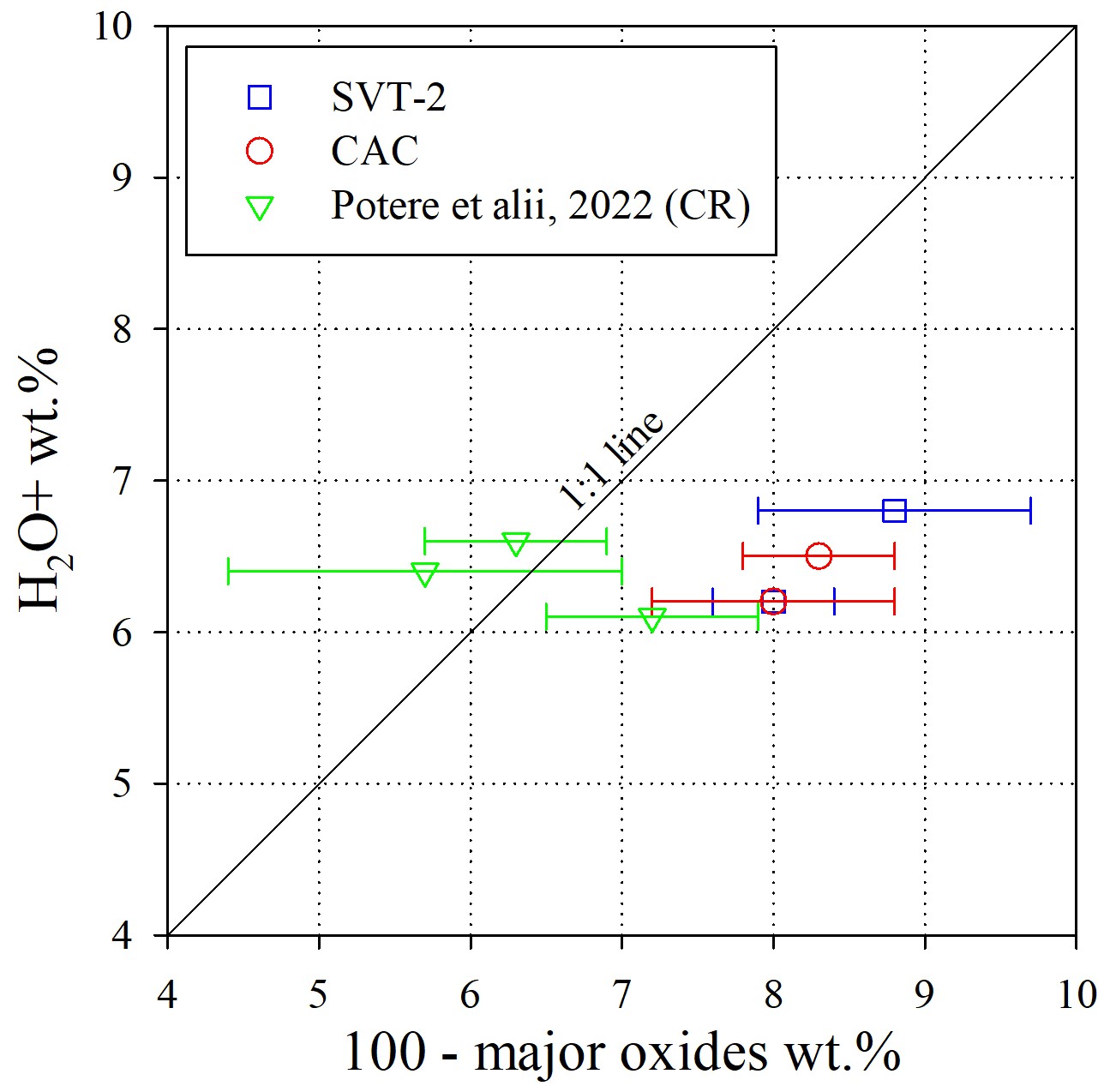

Supplement: Supplementary file 6 — Supplementary Information 6. [file 41598_2023_33256_MOESM6_ESM.jpg]

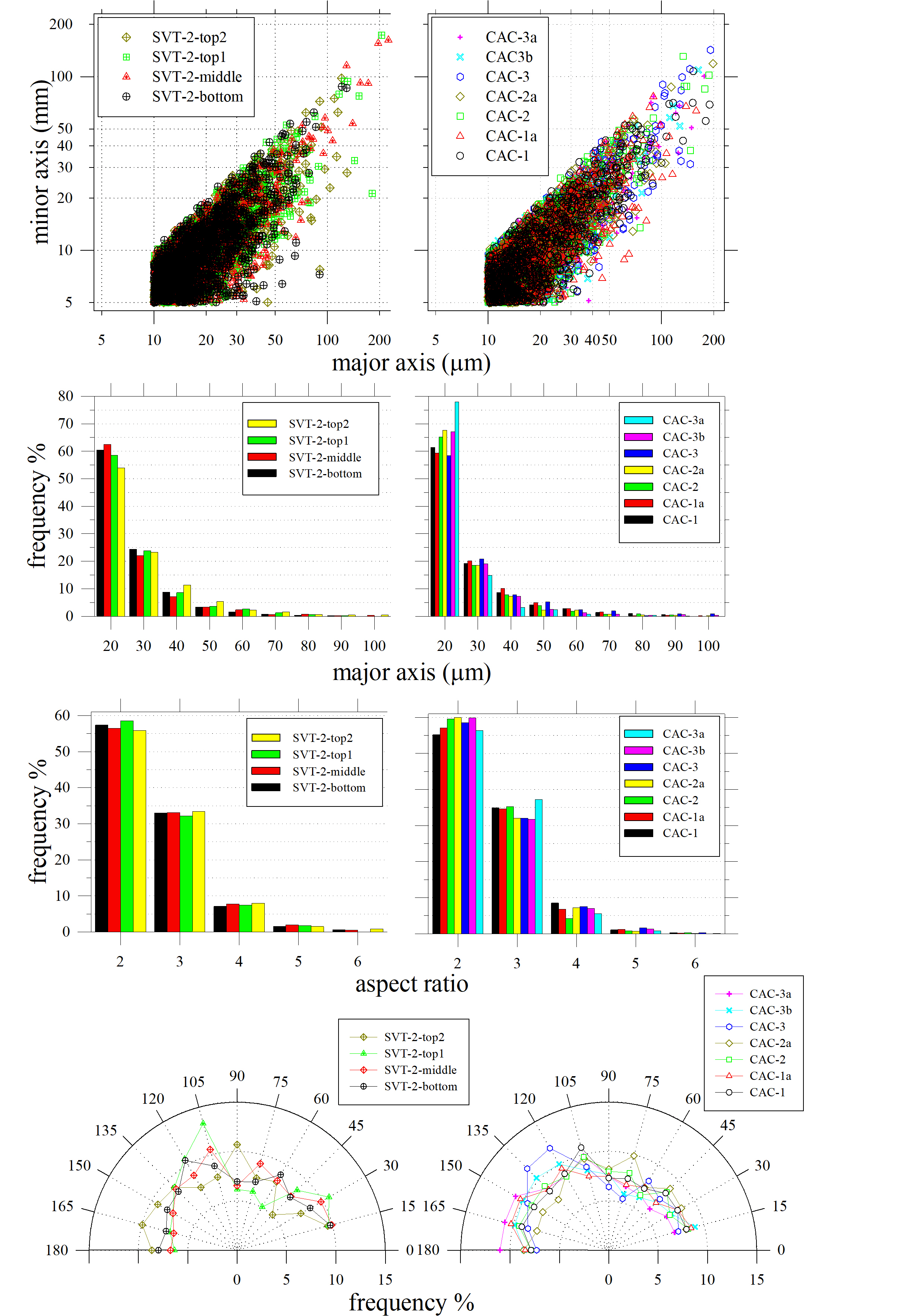

Supplement: Supplementary file 7 — Supplementary Information 7. [file 41598_2023_33256_MOESM7_ESM.jpg]
