# Supplementary material for: Provenance and deposition of a lithified volcanic-rich layer (VRL-5.5) at 5.5 Ma from Central Apennines (Italy)
Source: Sci Rep. 2023 Apr 27;13:6880. doi: 10.1038/s41598-023-33256-2 (PMC10140287; doi:10.1038/s41598-023-33256-2)
Supplement: Supplementary file 9 — Supplementary Information 9. [file 41598_2023_33256_MOESM9_ESM.pdf]

Supplementary (S) figures captions

Fig. S1 An enlarged, complete and detailed field view of the CAC outcrop and related specimens. The red box indicate the location of the stratigraphic section used of observations and sampling. The lower portion of it is reported in Fig. 2a

Fig. S2 Example of image analysis on two representative samples. From left to right columns: as-acquired BS-SEM microphotographs, segmentation in false colours and textural measurements of single objects. The false colours red and blue corresponds to glass and carbonates, respectively (middle column). The measured textural parameters are lengths of major and minor axes, aspect ratio (major axis/minor axis), area, perimeter, the angle between the major axis and the horizontal direction and roundness (right column). SVT-2-top1 is mainly glassy with a limited amount of carbonates, while CAC-3 shows a larger fraction of carbonates. The blue bars correspond to 20 μm

Fig. S3 Detail of the SVT-2-top2 sample, with XRPD spectra (left) collected on the different portions of the specimens (right) corresponding to the points 1, 2, 3. The "SH" pattern at the bottom is relative to the glass sample holder, which is therefore not zero-background. Recognised crystalline phase are anorthite (AN), biotite (BT), calcite (CLC), clinopyroxene (CPX), dolomite (DOL), illite (ILL), montmorillonite (MNT), quartz (QZ) and sanidine (SND). The red bar corresponds to 1 cm

Fig. S4 Detailed textural features of glassy ash grains (dark grey) and carbonates (light grey phases in CAC-3, SVT-2-top1 and SVT-2-top2) imaged by BS-SEM at different magnification. Red bars are 50 □m

Fig. S5 (top) Relationships between bulk SiO_2 vs CaO and CO_2 (from the data in Tab. S3), plus their linear regressions. (bottom) Variations of bulk contents of Stot, CO_2 H_2O^-, H_2O^+ and LOI. The data labelled CR refer to the Camporotondo section in Potere et al. 2022, see Fig. 1

Fig. S6 Differences respect to 100 wt.% of oxide total amount determined by EPMA (Tab. S6) compared with bulk determinations of H_2O^+ (Tab. S3). The data labelled CR refer to the Camporotondo section (Potere et al. 2022, see Fig. 1)

Fig. S7 Top row: major vs minor axes (log scale) of all equal-area ellipses of glass shards, quantified by image analysis on BS-SEM images at 200x; second row from top: major axis frequency distributions divided in 9 classes every 10 µm; third row from top: aspect ratio frequency distributions divided in 9 classes; bottom row: 2D angular frequency distribution of major axis considering 12 classes, every 15°

Supplementary (S) tables captions

Tab. S1 SVT-2 and CAC sample locations, labels and thicknesses

Tab. S2 Sites of VRL-5.5 reported in previous studies

Tab. S3 Bulk compositions of selected SVT-2 and CAC, plus CR (Potere et al. 2022) samples (raw data)

Tab. S4 Grain-size parameters quantified by 2D image analysis of SVT-2 and CAC, plus CR (Potere et al. 2022) samples

Tab. S5 Trace elements from bulk analysis of SVT-2 and CAC, plus literature (where available) samples

Tab. SI6 Micro-chemical composition of SVT-2 and CAC glassy matrixes determined by EPMA compared with VRL-5.5 from previous investigations (raw data)
